# Supplementary material for: Au30(PiPr2nBu)12Cl6—An Open Cluster Provides Insight into the Influence of the Sterical Demand of the Phosphine Ligand in the Formation of Metalloid Gold Clusters
Source: Molecules. 2024 Jan 5;29(2):286. doi: 10.3390/molecules29020286 (PMC10819969; doi:10.3390/molecules29020286)
Supplement: Supplementary file 1 [file molecules-29-00286-s001.zip › molecules-2794137-supplementary.pdf]

Supporting information

Table of contents

1. Synthesis 3

1.1 Synthesis of $^{i}Pr_2{}^{n}BuPAuCl$ 1 3

1.2 Synthesis of $Au_{30}(^{i}Pr_2{}^{n}BuP)_{12}Cl_6$ 2 3

2. NMR Data 3

3. Crystallographic data 9

4. Quantum chemical calculations: 11

5 Referenzes 28

1. Synthesis

The reactions were done in an inert gas atmosphere performing standard Schlenk techniques. THF and Cyclopentane were pre-dried with sodium. $^{i}Pr_2{}^{n}BuP$ and $Au_{32}(^{n}Bu_3P)_{12}Cl_8$ were synthesized as described earlier by our group.[1][2]

1.1 Synthesis of $^{i}Pr_2{}^{n}BuPAuCl$ 1

321 mg (1mmol) (THT)AuCl is solved in 20 ml THF. 0,2 ml (1 mmol) $^{i}Pr_2{}^{n}BuP$ is added. The solution becomes colourless. The solvent is removed after one hour.

1.2 Synthesis of $Au_{30}(^{i}Pr_2{}^{n}BuP)_{12}Cl_6$ 2

406 mg (1 mmol) $^{i}Pr_2{}^{n}BuPAuCl$ is suspended in 20 ml ethanol. 38 mg (1mmol) NaBH4 is suspended in ethanol and added quickly to the suspension, which turns black immediately. The ethanol is removed after one hour and the black solid is extracted with 20 ml cyclopentane. The extract is stored at 6 °C. If there is oil, the extract is filtered again. After a few weeks, the $Au_{30}(^{i}Pr_2{}^{n}BuP)_{12}Cl_6$ cluster crystallizes in form of black rhombus.

2. NMR Data

Devices: Bruker AvanceII+400 ($Au_{30}(^{i}Pr_2{}^{n}BuP)_{12}Cl_6$)

Bruker AVIIIHD-300 ($^{i}Pr_2{}^{n}BuPAuCl$)

The chemical shifts are given in ppm against the external standards $SiMe_4$ (1 H, 13 C,) and 85% phosphoric acid (31 P). C_6D_6 and $CDCl_3$ was dried with 3 Å molecular sieves.

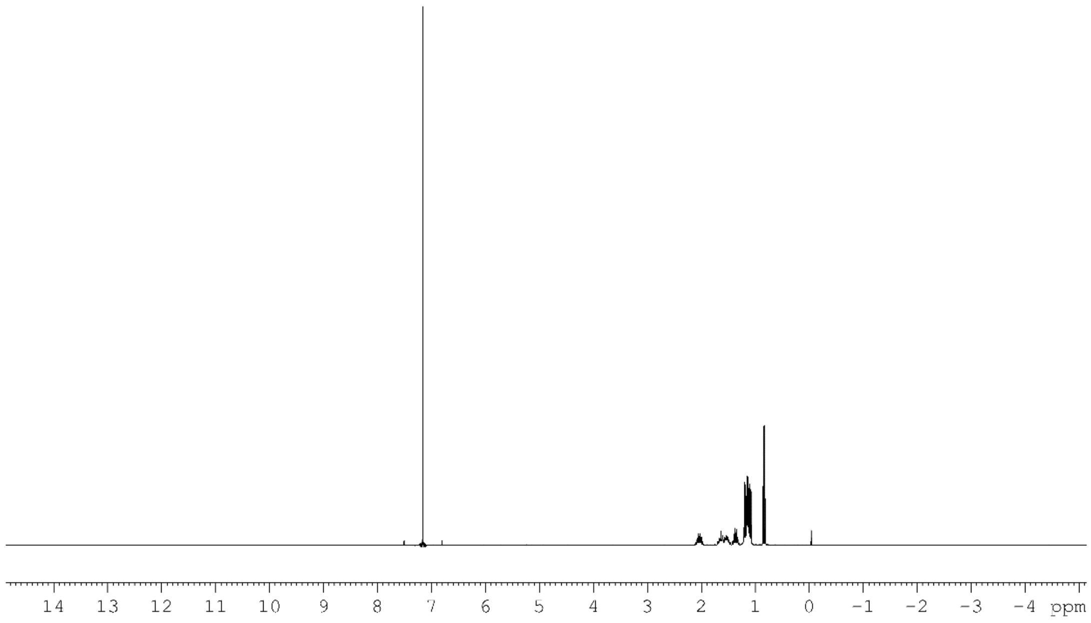

Figure S1: ^{1}H NMR-spectrum of **1** at RT in $CDCl_3$

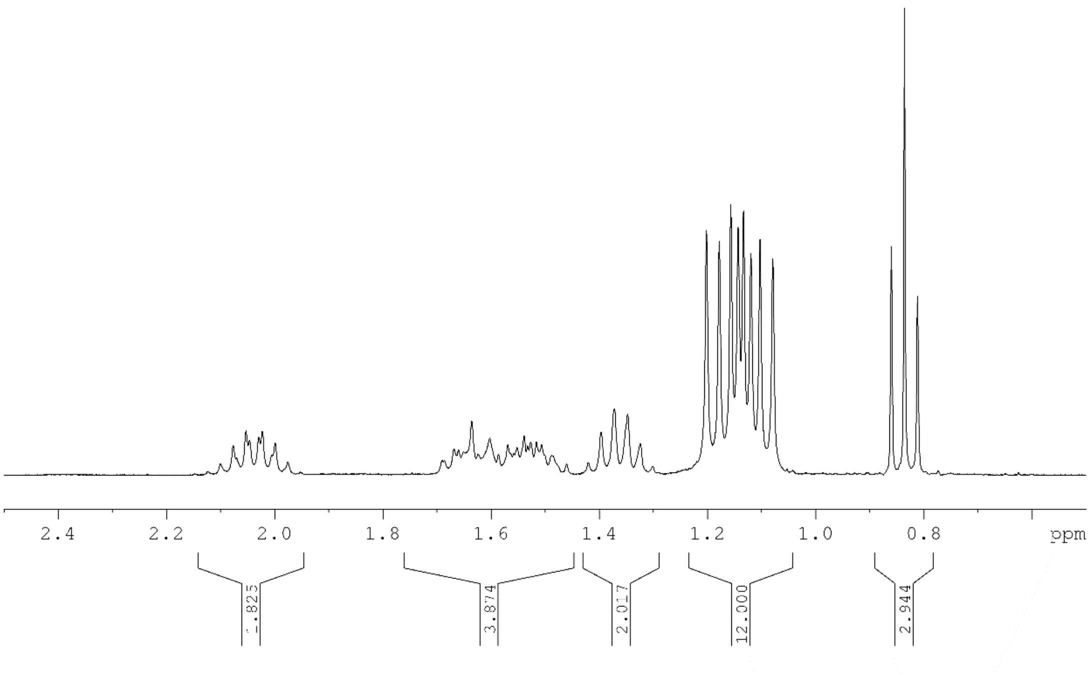

Figure S2: ^{1}H NMR-spectrum of **1** at RT in $CDCl_3$, the section between 0.5 and 2.5 ppm is enlarged

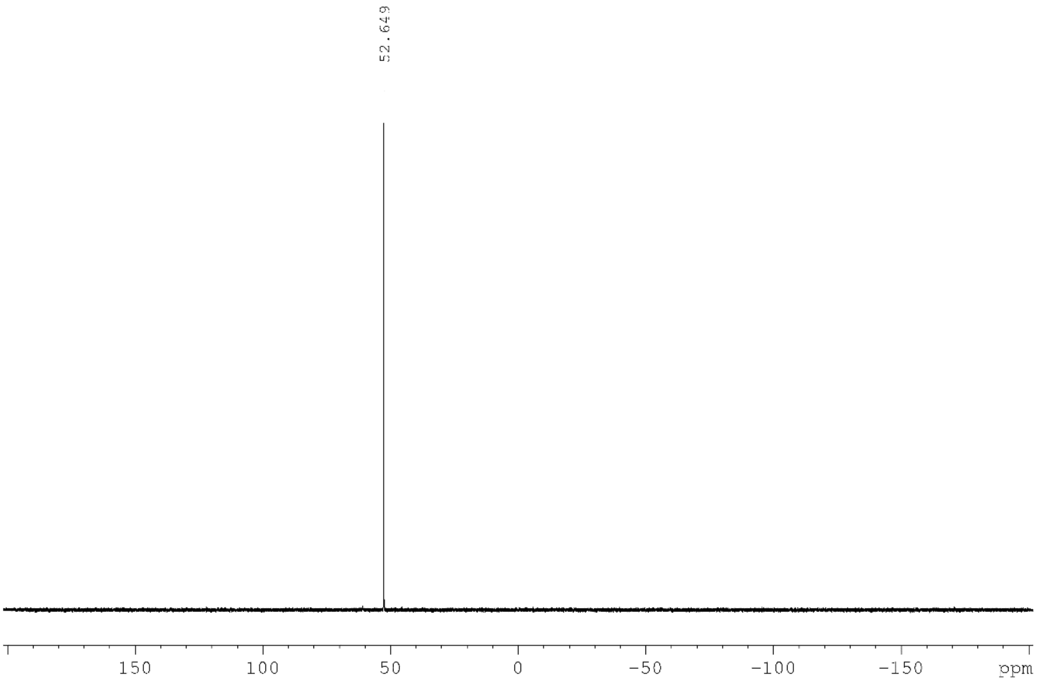

Figure S3: ^{31}P NMR spectrum of **1** at RT in $CDCl_3$

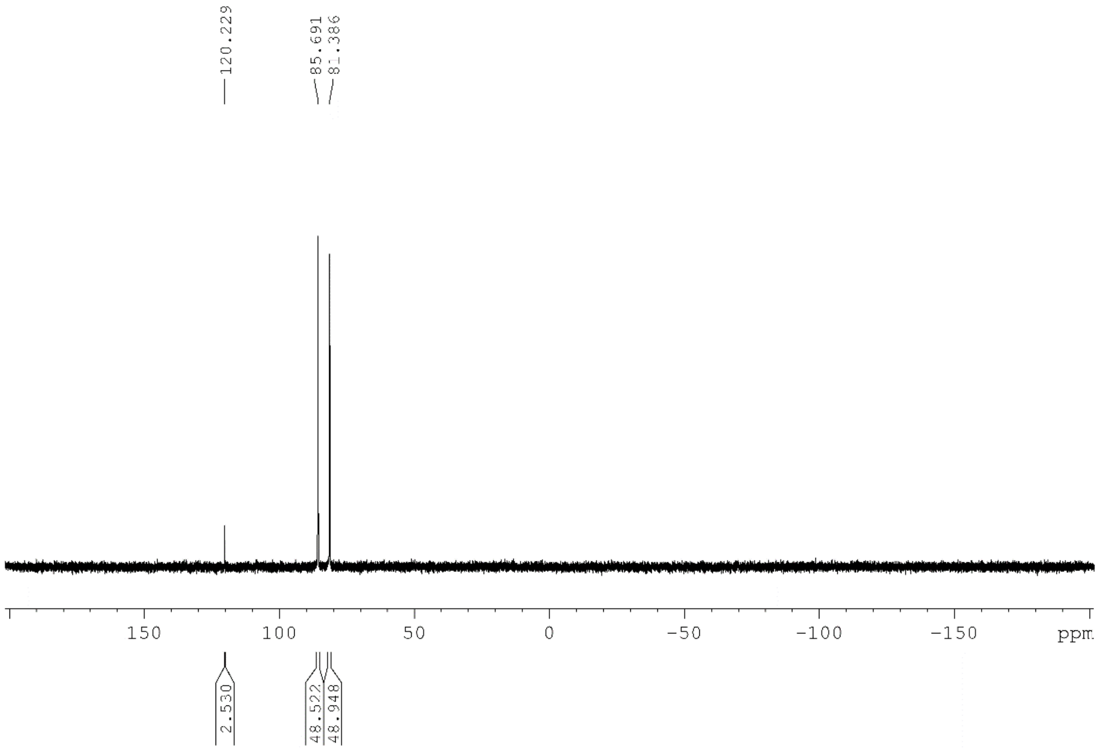

Figure S4: ^{31}P NMR spectrum of **1** at RT inC_6D_6

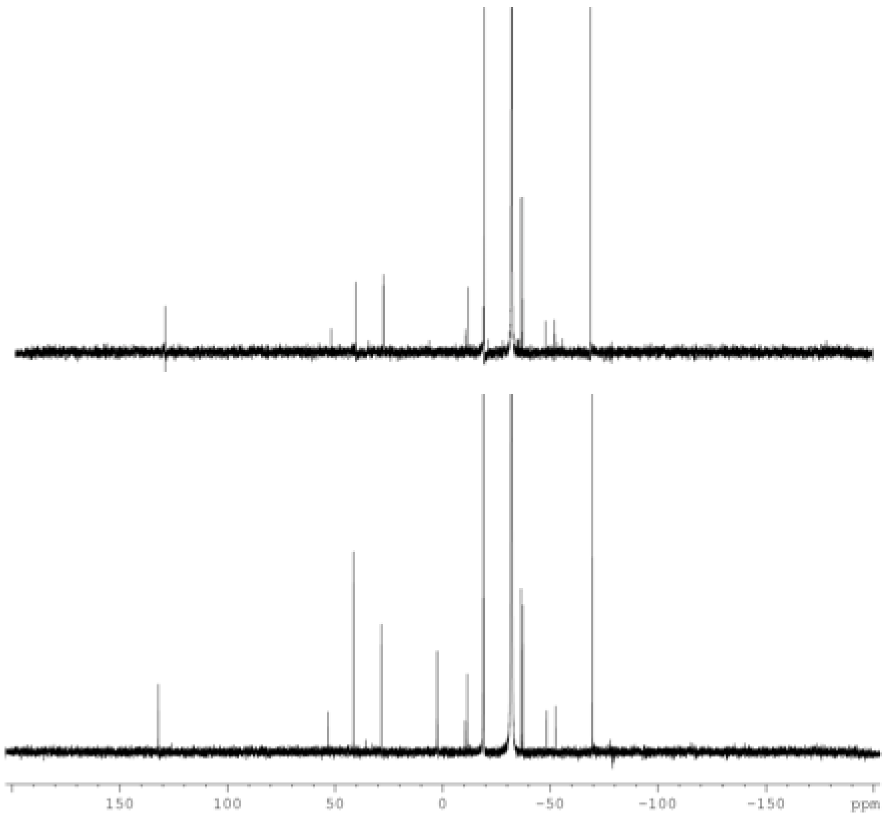

Figure S5: ^{31}P NMR spectrum of PBu_3 (up) and PBu_3 + **1** (bottom) in C_6D_6.

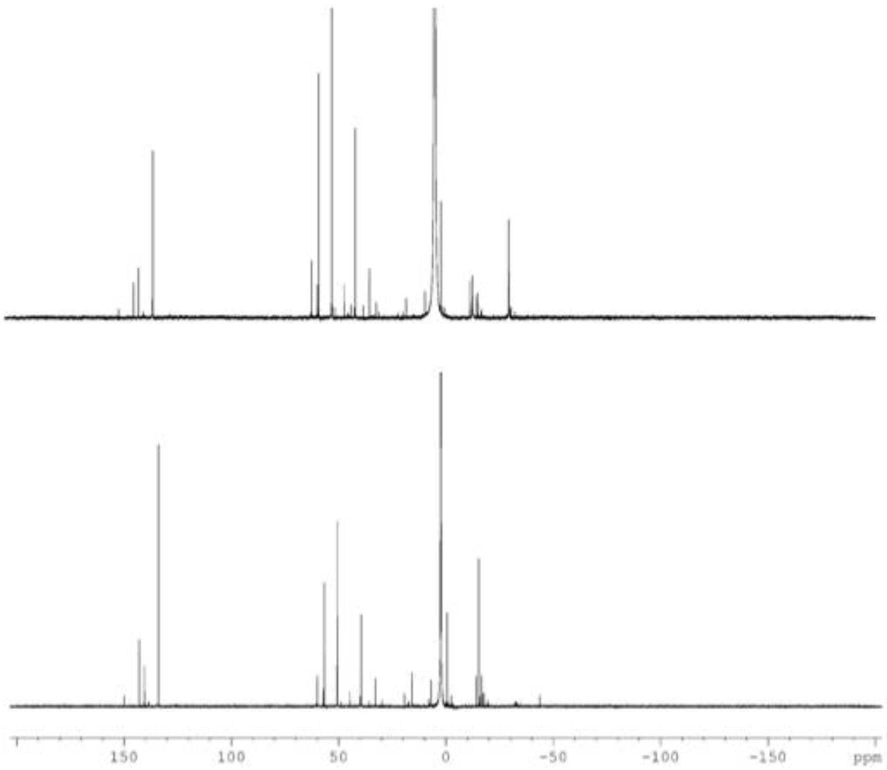

Figure S6: ^{31}P NMR spectrum of **2** + $P^{i}Pr_2{}^{n}Bu$ (up) and $P^{i}Pr_2{}^{n}Bu$ in C_6D_6.

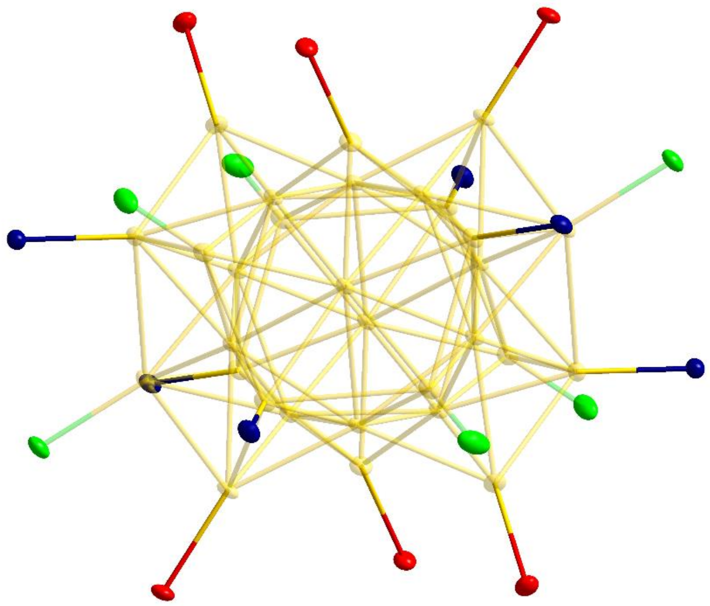

Figure S7: Core of **1** based on the crystal structure (gold: yellow; chlorine: green; phosphorus: blue, red (based on the chemical surrounding))

3. UV/Vis

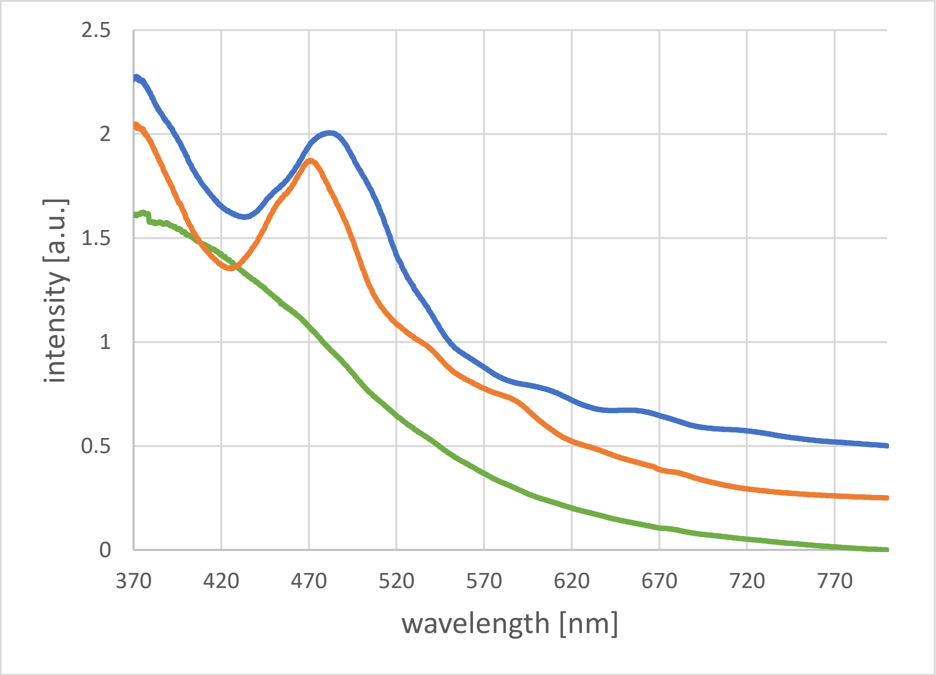

Figure S8: UV/Vis spectra of **1** (orange), **3** (blue) and **3** + $^{i}Pr_2{}^{n}BuP$ (green) in benzene.

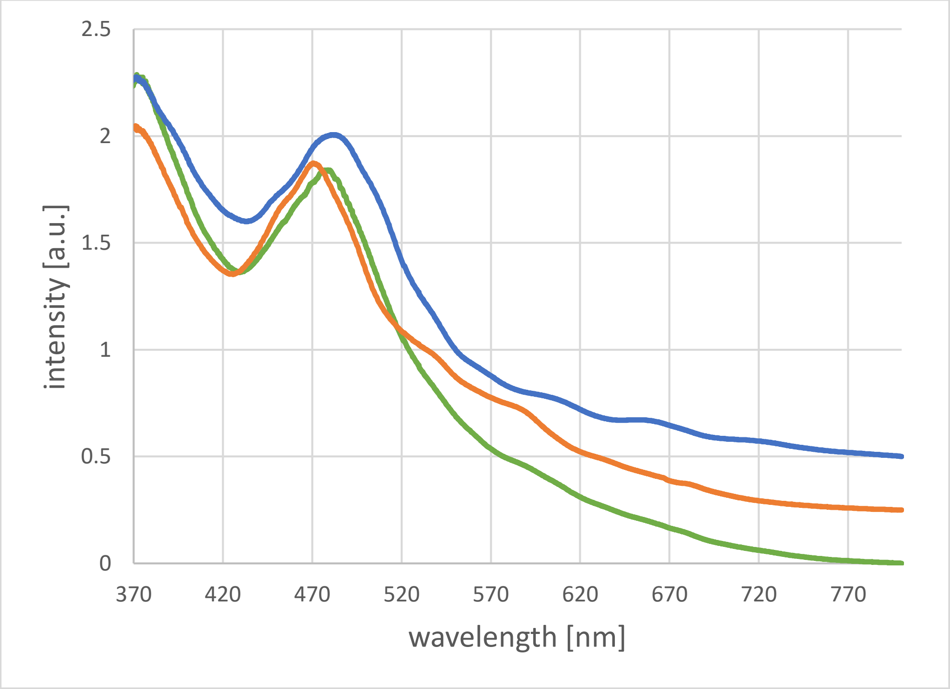

Figure S9: UV/Vis spectra of **1** (orange), **3** (blue) and **1** + $^{n}Bu_3P$ (green) in benzene.

4. Crystallographic data

Crystals were mounted on the diffractometer at 100 K. The data were collected on a Bruker APEX II DUO diffractometer equipped with an IμS microfocus sealed tube and QUAZAR optics for monochromated MoKα radiation (λ = 0.71073 Å) and equipped with an Oxford Cryosystems cryostat. A semiempirical absorption correction was applied using the program SADABS. The structure was solved by direct methods and refined against F2 for all observed reflections. Programs used: SHELXS and SHELXL[3] within the Olex2 program package. [4]

$^{i}Pr_2{}^{n}BuPAuCl$ **1**

CCDC Number:	
Empirical formula	$C_{10}H_{23}AuClP$
Formula weight	406.67
Temperature/K	100
Crystal system	monoclinic
Space group	P21/c
a/Å	9.6698(3)
b/Å	8.4076(3)
c/Å	16.4655(5)
α/°	90
β/°	91.9380(10)
γ/°	90
Volume/Å^3	1337.88(8)
Z	4
ρ_{calc}g/cm^3	2.019
μ/mm-1	11.277
F(000)	776.0
Crystal size/mm^3	0.461 × 0.194 × 0.056
Radiation	MoKα (λ = 0.71073)
2Θ range for data collection/°	4.95 to 59.998
Index ranges	-13 ≤ h ≤ 13, -11 ≤ k ≤ 11, -21 ≤ l ≤ 23
Reflections collected	20898
Independent reflections	3892 [R_{int} = 0.0363, R_{sigma} = 0.0304]
Data/restraints/parameters	3892/0/123
Goodness-of-fit on F_2	1.041
Final R indexes [I>=2σ (I)]	R_1 = 0.0182, wR_2 = 0.0422
Final R indexes [all data]	R_1 = 0.0207, wR_2 = 0.0437
Largest diff. peak/hole / eÅ^{-3}	1.03/-2.56

$Au_{30}(P^{i}Pr_{2}{}^{n}Bu)_{12}Cl_{6}$ **2**

CCDC Number	
Empirical formula	$C_{120}H_{276}Au_{30.06}Cl_{6}P_{12}$
Formula weight	8224.54
Temperature/K	100
Crystal system	monoclinic
Space group	P21/n
a/Å	18.5234(9)
b/Å	19.3804(8)
c/Å	24.5820(11)
α/°	90
β/°	96.1050(10)
γ/°	90
Volume/Å^3	8774.7(7)
Z	2
ρ_{calc}g/cm^3	3.113
μ/mm-1	25.248
F(000)	7305.0
Crystal size/mm^3	0.286 × 0.177 × 0.028
Radiation	MoKα (λ = 0.71073)
2Θ range for data collection/°	3.586 to 49.426
Index ranges	$-21 \le h \le 21$, $-22 \le k \le 22$, $-28 \le l \le 28$
Reflections collected	109202
Independent reflections	14950 [R_{int} = 0.0428, R_{sigma} = 0.0235]
Data/restraints/parameters	14950/18/762
Goodness-of-fit on F_2	1.054
Final R indexes [I>=2σ (I)]	R_1 = 0.0292, wR_2 = 0.0785
Final R indexes [all data]	R_1 = 0.0346, wR_2 = 0.0836
Largest diff. peak/hole / eÅ^{-3}	2.71/-2.38

5. Quantum chemical calculations:

All quantum chemical calculations have been done on a DFT level with the B-P86 functional (exchange: LDA + Becke (B88); correlation: LDA (VWN) + Perdew (P86)). The def-SV(P) basis set and additional for the gold atoms, def-ecp was used.

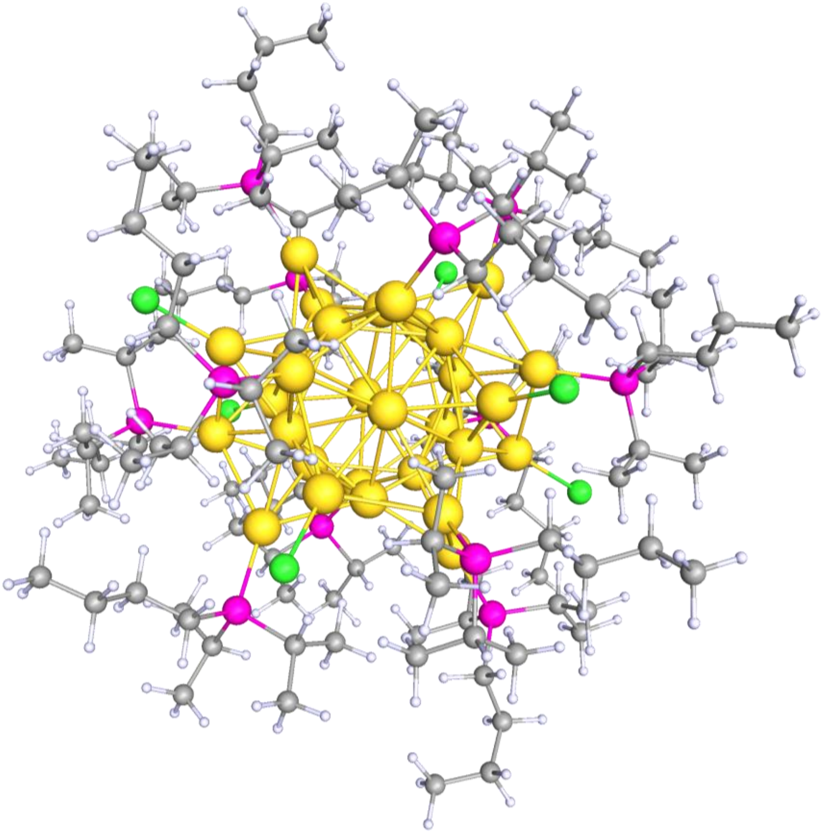

Figure S10: Geometry optimized structure of **2**

Point group: C_1

Energy: -15669.96961061505 H

HOMO-LUMO-Gap: 0.869 eV

Atomic coordinates:

1 au 3.31597561173539 36.86894301019531 4.17861777406424	2 au -2.15699388452618 36.32463717495765 5.01109412255903
3 au 0.88642086853421 32.09903277073298 3.13907892400418	4 au -0.07400038794539 41.05972971141510 2.39325419208094
5 au 4.12170171232049 39.65414427929019 -0.77728339134599	6 au 4.79964705676733 34.19450802639857 -0.60693953830403
7 au 8.42647455088144 37.42219381681051 1.67472040696308	8 au 0.80786047599077 39.87919008347568 7.84255069473764
9 au 1.26115160515792 34.16336351479666 8.47242797002129	10 au 4.97881550669558 41.87549853028280 4.17623817322864
11 au 6.31673763518837 32.37528006252408 4.09531221783939	12 au -4.13711868452662 32.89677542755150 0.83596714187726
13 au -4.82841278993814 38.36116098293484 0.69025843390446	14 au -7.52781276278237 34.97159218470762 4.21533193826681
15 au -4.58897384041868 41.27219897021203 5.17455990693119	16 au 0.05430802738412 31.49532426204032 -2.33268045122360
17 au 3.81119724407742 28.62967829321482 0.20914208503837	18 au -1.74949666455577 27.84502992717249 1.38670604773024
19 h -0.58982847751183 28.82295103283428 7.58005564202422	20 au -0.91147440317564 40.46389924962403 -3.07350951650906
21 au -3.82939068391066 43.93389890174657 -0.14560357609056	22 au 1.72883264058269 44.71566339480822 -1.32639210062942
23 au 2.12224632070599 36.23529839714933 -4.93717938204746	24 au 7.50147193860853 37.49931696901680 -4.15040870166590
25 h 7.84524413371321 43.62576139340917 -1.85977207853159	26 au 4.57566518140911 31.31634800894177 -5.10981648880744
27 p 12.19443780944779 37.77295540430354 4.08073428471472	28 p 0.67004917617662 43.10733861166953 10.93878677843884
29 h -1.37065916649981 45.61131667225113 6.40427529679382	30 p 2.45773955869323 31.15927849163177 11.66937129740472
31 h -1.67566135373128 34.55201241849461 13.34867323672865	32 cl 7.78900669300666 45.27557043689794 5.73540270517064

33 cl	8.94296786634786	29.97051649042399	7.13857653713317	34 h	9.80769094967756	27.84956149920709	2.42028703957128
35 au	-3.34438709146366	35.69085327081273	-4.10599203324545	36 au	-8.44818729166506	35.12417863923610	-1.59714805673510
37 au	-4.99915117776784	30.67885709817854	-4.11831994058442	38 h	-7.87293110452964	28.93747669780362	1.90065951148981
39 au	-6.34879234967270	40.17951526268079	-4.00985838551638	40 p	-10.26586628560157	35.03687509716986	7.79809236636910
41 cl	-7.69503107072337	43.38525843793629	8.04779325382141	42 au	-0.83749944278920	32.67993467337133	-7.77428568639591
43 p	6.93468397897869	25.44604205601074	-0.09300196776372	44 h	4.36950825641606	24.73633023905651	5.01329491786156
45 p	-4.45732226179301	24.26805831297048	1.90481130193587	46 c	0.05729355081582	27.29840618397302	8.87649504296946
47 au	-1.28698580447167	38.39605087578741	-8.40223096056816	48 h	0.58504064296630	43.72878571925465	-7.52296563563127
49 p	-6.94977208628933	47.12124499305543	0.15090411003940	50 h	-4.38772282003450	47.81633635026783	-4.95910426771044
51 p	4.43430263613579	48.30009217276007	-1.82023193244508	52 p	10.26289646666507	37.59264167428152	-7.71528890939815
53 c	8.91421408801092	45.43993857658300	-1.93108669065208	54 cl	7.68423437615231	29.22024319897444	-7.97209339592970
55 h	1.36096025017484	26.95881171554556	-6.33391769735166	56 c	11.72024167985725	36.64579766884663	7.42261941225499
57 c	14.89487320299428	35.78206690042209	2.86003722216090	58 c	13.45334528485467	41.02976905284959	4.54916076467441
59 c	3.02449269824856	42.71544824334179	13.56514766271621	60 c	-2.47552381098974	43.25008266821377	12.49382604395495
61 c	1.36971877525928	46.28028637208525	9.47620207091848	62 c	-0.85318727779164	47.10341600402353	7.79513711489949
63 c	2.59578787256687	27.94930722983247	10.12316858958409	64 c	5.65029539918042	31.48349462021574	13.10004590188292
65 c	0.12295864735760	30.93586799676998	14.37690731333856	66 c	-0.95848472644458	33.55089125590729	15.04963621954965
67 c	10.17172536563970	26.70059789416644	0.69084298488747	68 p	-12.21875124661371	34.78041842213272	-4.00121365560100
69 cl	-7.81586361354069	27.27594017627529	-5.65769727268555	70 c	-8.94093549101759	27.12427438947929	2.00428297563834
71 cl	-8.95970520639833	42.58539507535134	-7.06600550751755	72 h	-9.82968275607069	44.71777020878288	-2.35336315945063
73 c	-8.55212445550253	34.86655978575663	10.90125485294055	74 c	-12.46367488369952	32.21803131283670	7.84030739038860
75 c	-12.17646540685265	37.98986513439613	7.87081599719056	76 p	-0.69527200001542	29.45084689683443	-10.86831594294905
77 c	6.39546923403060	22.67295460986205	2.05944813077505	78 c	7.00170725856265	24.13478446052779	-3.35156294778457
79 c	6.10518369687896	23.56657570827646	4.80850974626174	80 c	-5.36339803724605	22.74979813708049	-1.17994018290324
81 c	-2.98551063744317	21.79610173860624	3.95774087091599	82 c	-7.48601453419057	25.13190934057363	3.53722728756176
83 h	0.27551750541123	25.55782623841359	7.71566097689814	84 h	-1.45321425223346	26.92733147537324	10.29556772812283
85 p	-2.47663116935078	41.39343933998212	-11.60609280714298	86 h	1.62372111978853	37.96299850068612	-13.28688565968101
87 c	-0.05294162024732	45.25084069730906	-8.82677698550163	88 c	-10.18977439736260	45.86900006019854	-0.62462205222217
89 c	-6.41103996878075	49.88846278172007	-2.00956154573891	90 c	-7.00803535026609	48.44051715956028	3.40634034871769
91 c	-6.12250399425716	48.98771441673087	-4.75657320122808	92 c	5.32774011827544	49.80675100345516	1.27441102755354
93 c	2.97034133166304	50.78268224984781	-3.86636502919469	94 c	7.47145081299578	47.45439067399186	-3.44652228527647
95 c	8.56215745387649	37.76471707700027	-10.82691706765549	96 c	12.29004555759721	40.53985785360267	-7.64737302390457
97 c	12.36231310553651	34.76831603614360	-7.86216001631739	98 h	9.27850776404704	46.00986949065665	0.05627392530250
99 h	10.76716991607691	45.06076067368571	-2.85363592173519	100 c	0.84514123529436	25.46328814026789	-7.72166269618900
101 c	9.54302621305608	38.08103379014725	8.69419632594822	102 h	11.10848198938228	34.64364619209633	7.18609744124435
103 c	14.12726580402254	36.71039770957517	9.05718704933411	104 c	14.48509155153342	32.96915855368093	3.46525175836412
105 c	15.27616991012863	36.15640934088786	0.00473008433853	106 h	16.60982026924936	36.47634715353045	3.87125888528517
107 h	11.84513744330700	42.09880643216275	5.39542137074699	108 h	14.97062398856678	40.91027564924429	6.00403315966181
109 c	14.42121807536264	42.49748734748323	2.24019622207404	110 h	2.87491432969736	44.46057339959951	14.73386816474084
111 c	5.71395058835768	42.50457308439719	12.49213463143047	112 c	2.35586840571939	40.42628891592818	15.22221221538302
113 h	-3.88372008050770	43.44727251978340	10.93772907503497	114 h	-2.77972921596364	41.30858899995236	13.24778700143542
115 c	-2.98115023360592	45.22983324615999	14.56246529380385	116 c	2.18750669956278	48.38005549392080	11.31625443122396
117 h	3.00809958573614	45.85487097961757	8.22022255941020	118 h	-2.57422738828540	47.55991553010988	8.91151621837531
119 h	-0.30890124613922	48.82341123232112	6.71444867077501	120 h	4.00737293381697	28.32306635043225	8.60170285506571
121 c	3.59138489018769	25.74439706706245	11.73355752008575	122 h	5.92658631076656	29.88236678479078	14.43771522396680
123 h	6.97677679736364	31.17915083981232	11.48637301669657	124 c	6.22276704570332	33.99561004426611	14.43624159410342
125 h	-1.44015159549065	29.79313898088432	13.54625855867366	126 c	1.10946397227158	29.57394516850333	16.75092876451758
127 h	-2.54057740221749	33.34784496135111	16.42446191562546	128 h	0.49535671326266	34.77766609512413	15.94189681104975
129 c	12.18426178961167	24.71166695206162	1.36395573359322	130 c	11.04679543221906	28.49998554741510	-1.41796322219687
131 c	-11.74815058819866	35.90047360440354	-7.34584390860376	132 c	-14.91152932463854	36.77969345093043	-2.77795817474242
133 c	-13.48691344427201	31.52547428593561	-4.45985703965912	134 h	-9.32132508583902	26.52695170767412	0.02816403539910
135 h	-10.78573429264003	27.51678086387721	2.93761856891744	136 h	-10.05298865002843	34.92562690905189	12.37887262640640
137 c	-6.79408425834898	37.14826913157381	11.26609266203811	138 c	-7.09330882035039	32.36174981912701	11.13069089056195
139 c	-14.19417371166674	32.26097423236240	5.51109556568959	140 h	-11.11717163455658	30.61771518356074	7.58922284051087
141 c	-13.96344732501359	31.77046710191556	10.29082831392640	142 h	-10.76943409367392	39.52239307369657	7.52011493122020
143 h	-13.37881769677097	37.92017027220993	6.14410598110106	144 c	-13.76599026272263	38.66105709731662	10.21276985917730
145 c	-3.05679266795130	29.83246213441643	-13.49003077080786	146 c	2.44728438080492	29.31588626680256	-12.43149052461345
147 c	-1.38229699465266	26.27734241682240	-9.40078936812688	148 h	8.11539030627054	21.46929933880326	1.90044909724028
149 c	4.09620709789775	21.12756413918990	1.18815940928171	150 h	5.02893129928944	23.49496479734168	-3.71098945457653
151 h	7.27440916280223	25.79215811542874	-4.62625680831667	152 c	8.89142701270691	22.03157340099595	-4.02943014479414
153 h	7.70864532794867	24.75168522854330	5.46750460956134	154 h	5.94625372568828	21.90273365625771	6.08982760935632
155 h	-5.81891255660139	24.41404686562922	-2.39454230904923	156 c	-7.70906182716686	21.03363729394280	-1.16703783583236
157 c	-3.04835275372521	21.42950565122404	-2.33910225627869	158 h	-1.23206977958814	21.20861424099699	2.95743461950195
159 h	-2.32487460849587	22.85133718610441	5.65127155194397	160 c	-4.53226185132255	19.45652391821650	4.73081796261492
161 h	-8.62383492327777	23.36082373650551	3.62158094649297	162 c	-6.97445022251476	26.05019655015313	6.24685429619230
163 c	-2.59590044818755	44.60924908656582	-10.06955858737414	164 c	-5.67376222474672	41.07960840744636	-13.02827910051695
165 c	-0.14557867144876	41.59398974752831	-14.31839440117519	166 c	0.91614643581603	38.96999138506710	-14.98858529608543
167 h	-0.25945801541025	46.99870291998755	-7.67456833459955	168 h	1.45930405978938	45.60498157275698	-10.24828372810759
169 c	-12.20244875196250	47.85817542488974	-1.29619508293261	170 c	-11.06146671512796	44.07241472751967	1.48778431969905
171 h	-8.13043730800886	51.09316656679252	-1.85294397871566	172 c	-4.11078969378955	51.43527939856001	-1.14359098155883
173 h	-5.03271553578657	49.07503792002293	3.76109580364767	174 h	-7.28292884304309	46.78635273351613	4.68464863759353
175 c	-8.89010904270478	50.55036600105662	4.08424517306573	176 h	-7.72698274170691	47.80182580380976	-5.41166257982255
177 h	-5.96322056454918	50.64811618457964	-6.04224499655428	178 h	5.76895181759631	48.13833858148405	2.48828947889505
179 c	7.68179721173532	51.51141013261942	1.28026920490906	180 c	3.01102394870539	51.13528128243104	2.42075233788900
181 h	1.20645415624151	51.35700369505614	-2.87731232867924	182 h	2.32783707459710	49.73846495670412	-5.57368800226493
183 c	4.51744923020630	53.13305888678430	-4.60627972350198	184 h	8.61176821834439	49.22491964076703	-3.49864589491207
185 c	6.97679721148912	46.57718047505890	-6.17268065911692	186 h	10.07003063261383	37.88762167977426	-12.29375068939705
187 c	6.97298216133829	35.38475478575823	-11.31471782162690	188 c	6.91924356869810	40.16200138749789	-10.94440318150351
189 c	14.12991278499965	40.42609032030624	-5.40545652429108	190 h	10.86496680340377	42.02551790976861	-7.20460206941387
191 c	13.62687619257845	41.28861182123700	-10.12028366421747	192 h	11.02995082483916	33.13404819208366	-7.91382275289495
193 h	13.27918980962786	34.67131695749328	-5.96977440537523	194 c	14.37232710056987	34.58336518549598	-9.96115303052384
195 h	2.56530977554550	25.00888110611188	-8.84034312119426	196 h	0.30693075495623	23.74370179925129	-6.63712940867465
197 h	7.75096298014151	37.89301360564367	7.61005123463332	198 h	9.22003941521185	37.28034119904828	10.61128326019436
199 h	9.94998911452168	40.13026013699006	8.91080504924376	200 h	15.76158974143986	35.73321626053466	8.17073325925570
201 h	14.71300628354237	38.67540205885069	9.52173514526642	202 h	13.74379064017010	35.73527841529967	10.88256607511500
203 h	14.48705054812790	32.54635352136249	5.51725474889421	204 h	12.64720541739344	32.29433320318380	2.70376002671219
205 h	16.01803818807509	31.83186385966109	2.57660210583015	206 h	15.70092360924405	38.14584629389046	-0.49501554540648
207 h	16.88413833823379	34.97082797061991	-0.66115858096193	208 h	13.55129903806386	35.58104392791920	-1.05293902029962
209 c	15.04879260068252	45.25792011598853	2.91875894916722	210 h	12.96411162985814	42.45884578529489	0.72094655257397
211 h	16.14610519472752	41.58007367167978	1.45299793040040	212 h	6.24273874834862	44.10881632435231	11.24505321140562
213 h	7.10950478329980	42.41446010713747	14.06594499018755	214 h	5.90481251288333	40.75805784062528	11.34164439112825

215 h	0.51801966813838	40.65635279704597	16.21225566810026	216 h	2.26247548940730	38.67291501119993	14.06496908965625
217 h	3.83726835616433	40.15128412047260	16.69171083367221	218 h	-2.86463412108812	47.16259006972429	13.73931724516531
219 h	-1.51184759962073	45.14326915913422	16.07223778458501	220 c	-5.61281059138049	44.88893670247512	15.76009750958792
221 h	3.92163350718635	47.88186207668132	12.38854996255661	222 h	2.63659218648581	50.11402281357539	10.21000531461348
223 h	0.68284128613487	48.88537587107206	12.69150999467480	224 h	5.44007767774056	26.16393059489765	12.63293909906709
225 h	2.22949541210252	25.16783608024317	13.22769727904691	226 h	3.88590325988715	24.07208963101564	10.48990569766847
227 h	4.85766269485423	34.32497126503291	16.00674962362018	228 h	5.95224271397501	35.58706161335081	13.08560699123052
229 c	8.92340129323627	34.06181411035113	15.51768089388327	230 h	1.84846390380786	27.65391697068238	16.34879357856898
231 h	2.63789702356345	30.67065739178191	17.68833717893022	232 h	-0.45005505277865	29.37156976432285	18.15174033662890
233 h	11.64516347776397	23.55861085797834	3.03371758063840	234 h	12.60862062136629	23.40978883694915	-0.22848929130060
235 h	13.97485652821624	25.69939494575490	1.86320821731370	236 h	9.62241671220135	29.99985056742064	-1.79400484820327
237 h	12.83635931508899	29.43822478972479	-0.83767704222144	238 h	11.40375122504289	27.50176042173888	-3.23158435583751
239 c	-9.57682050002536	34.45793626432565	-8.61895272100153	240 h	-11.13161948114853	37.90165960421956	-7.11359615452635
241 c	-14.15876329570405	35.83872351977934	-8.97550849842975	242 c	-14.49803842731551	39.59082331455803	-3.38855304547145
243 c	-15.28390567333418	36.40990423757655	0.07908182983380	244 h	-16.63100650899591	36.08721505345498	-3.78295798380264
245 h	-11.88114537157531	30.44928352442449	-5.30178933914180	246 h	-15.00280812281271	31.64461747247567	-5.91619593561226
247 c	-14.46133712586218	30.06834844168915	-2.14689687917303	248 h	-7.77231547894465	38.99315694745559	11.05582349838680
249 h	-5.94642440702624	37.07249697966277	13.19094208637185	250 h	-5.22407786786801	37.12816268022561	9.86314908097641
251 h	-8.33211923441250	30.67103981083807	10.99186430974401	252 h	-5.61770809225658	32.22403179703681	9.63669960427087
253 h	-6.12573229073137	32.27966819788996	12.99619250795302	254 h	-13.08479431856452	32.51379368473421	3.74586596486480
255 h	-15.25625529516630	30.45036853193783	5.36121658256269	256 h	-15.61064945540820	33.81114599252794	5.62375717091049
257 h	-12.73710285680271	31.65252415260466	11.99071411294124	258 h	-15.38502454028141	33.27918016620917	10.62425054654338
259 h	-15.01026211792902	29.94828815471439	10.15086874988839	260 h	-12.56352744327017	38.59507286508251	11.94005312122399
261 c	-14.93088791993932	41.32038750970880	9.99471092773060	262 h	-2.90197558963220	28.08824241771623	-14.65944419935013
263 c	-5.74545194891209	30.03148390613822	-12.41253933038500	264 c	-2.40168490797036	32.12497031269653	-15.14775319979460
265 h	3.86134295294318	29.12172796161089	-10.88036120177002	266 h	2.74418888247418	31.25841480359009	-13.18590318010224
267 c	2.94850410716468	27.33836271733108	-14.50358898516471	268 c	-2.19560544834211	24.17296219666893	-11.23755835605232
269 h	-3.02045063952967	26.69932278048618	-8.14324661136316	270 h	4.39916826886507	20.21125677364232	-0.67654617803108
271 h	3.68683226911647	19.60335545049146	2.58182679792298	272 h	2.39237341732269	22.35363735447444	1.04394174393765
273 c	8.51136864264119	21.08088726686954	-6.75467131797509	274 h	10.85726182279541	22.75640375427270	-3.84109349495154
275 h	8.73257527837718	20.40708013497369	-2.69429000149357	276 h	-9.41293067513106	22.01083307861005	-0.42809277995501
277 h	-8.14726407038706	20.47720638917317	-3.14861130615743	278 h	-7.42772679781203	19.27050224129808	-0.05947125404664
279 h	-1.37251578911686	22.69839571597606	-2.36526706373174	280 h	-2.53293276146069	19.66926193839905	-1.30634101197354
281 h	-3.46906627518284	20.89291225336964	-4.32887741600678	282 h	-5.06553315824988	18.37433328011630	3.01108086006116
283 h	-6.34132487972380	20.04332298074090	5.63694408381806	284 c	-3.10852131199002	17.66281612675852	6.53993146042919
285 h	-6.05941407779012	24.58843067966380	7.44661205233920	286 h	-5.75189907784956	27.76142723842696	6.23978115325226
287 h	-8.79478536006930	26.56723147122626	7.16910616508494	288 h	-4.00798094199032	44.24707527781058	-8.54573008638469
289 c	-3.58097647248892	46.81474658672311	-11.68573591871756	290 h	-5.94658690696854	42.67765045271874	-14.37035629265987
291 h	-6.99508285350915	41.39370126569501	-11.41211949976903	292 c	-6.25970726602533	38.56548068955670	-14.35486527790128
293 h	1.42694096162399	42.72733404899024	-13.49247421918275	294 c	-1.12673531005126	42.95825877418864	-16.69330022240811
295 h	2.50102972292281	39.16022628141649	-16.36203911405369	296 h	-0.54663730848650	37.75498524726162	-15.88226454691508
297 h	-11.66501221213295	49.01118278910016	-2.96652258013783	298 h	-12.62514302861207	49.16021286694104	0.29664847018714
299 h	-13.99367492806260	46.87061472343510	-1.79355613894618	300 h	-9.63614207944823	42.57280974432297	1.86159822226869
301 h	-12.85218909811590	43.13353603389324	0.91226317124103	302 h	-11.41410264093487	45.07251489360387	3.30123119058437
303 h	-4.41132819856013	52.35484035946028	0.71989575520562	304 h	-3.70272383683999	52.95699086191049	-2.54032426829360
305 h	-2.40739565241581	50.20864726597738	-0.99918570229800	306 c	-8.50657073185575	51.49872871356452	6.80977960906529
307 h	-10.85865546853059	49.83303504126062	3.89534877585603	308 h	-8.72476529932458	52.17448990040236	2.74944250063064
309 h	9.38763016458297	50.52322593329441	0.56091834429707	310 h	8.10374830792928	52.06967864708393	3.26480278859171
311 h	7.42076905532548	53.27338589074560	0.16586407330005	312 h	1.32925888997531	49.87400661905919	2.43403206948493
313 h	2.51002299566357	52.89967511257977	1.38802718392824	314 h	3.42119428338410	51.66665915766497	4.41410613695041
315 h	5.00837779474198	54.21521201488173	-2.87384168648249	316 h	6.34869936355076	52.56016297797302	-5.47606064849576
317 c	3.11983297603209	54.92041906074762	-6.44180182777364	318 h	6.06274991525222	48.05457691508451	-7.35378157513697
319 h	5.76028841876352	44.86204399371259	-6.19981274845684	320 h	8.80373868706417	46.08452432893039	-7.09472902862809
321 h	8.08747076497064	33.61012154704638	-11.23160700174069	322 h	6.09657773681127	35.51460274215689	-13.22395964170587
323 h	5.42595254058156	35.20382891313127	-9.89927373389930	324 h	8.01622425233164	41.93063883037430	-10.66160010397091
325 h	5.40229982699608	40.09037772710806	-9.48704059977094	326 h	5.99399058556254	40.28951760385916	-12.82868758950026
327 h	13.11647098600056	39.98865862117619	-3.61910823109550	328 h	15.09840829144117	42.27886358814231	-5.16498208485053
329 h	15.62088900619395	38.97026022418416	-5.68902886154428	330 h	12.29836089156571	41.50447346379946	-11.73092283179682
331 h	15.09777001914034	39.90156305362222	-10.68126257741645	332 h	14.58737630047826	43.14456007636847	-9.85676850449395
333 c	15.82630828348567	32.05148942764788	-9.96624076975426	334 h	13.48634047482423	34.86475845399576	-11.85225071348395
335 h	15.76601039375351	36.13950603621139	-9.72494034307674	336 h	13.29806697374410	46.18857498149985	3.62621609026979
337 h	16.40867955810008	45.27675230941357	4.53067763304344	338 c	16.16400337467088	46.76801480105051	0.70935176625727
339 c	-6.23955482849503	46.89843768832631	17.75362780624388	340 h	-7.05591416866641	44.91728528870244	14.22766610065127
341 h	-5.73074893199377	42.97129346618642	16.62863871543171	342 h	10.29565901625778	33.78496145089719	13.94330544641739
343 h	9.18903124153816	32.41226160492037	16.80392995895583	344 c	9.54442648132029	36.50669966688621	16.94615960504499
345 h	-7.78249642212717	34.64295580719407	-7.53804969751730	346 h	-9.25520251813880	35.25492316995224	-10.53780812939346
347 h	-9.98928480842964	32.40939453664619	-8.83152455271284	348 h	-15.78832954831474	36.82215305297871	-8.08743313291492
349 h	-14.75077886821710	33.87434811937699	-9.43476726058528	350 h	-13.77680020219974	36.80893311274185	-10.80383050829921
351 h	-14.50394890789453	40.01076754891552	-5.44115174062513	352 h	-12.65724820726696	40.26355008773505	-2.63216969686626
353 h	-16.02681109131152	40.73207735645445	-2.49784147458127	354 h	-15.70554050051228	34.42101024235831	0.58293544072393
355 h	-16.89004132966649	37.59604728976154	0.74840534571549	356 h	-13.55587976861546	36.98655842107862	1.13076249542837
357 c	-15.09811045055149	27.30783489162943	-2.81706748468877	358 h	-13.00588713900968	30.10718297493320	-0.62600942450877
359 h	-16.18391939455345	30.99393804714230	-1.36457900650983	360 h	-6.26645549497458	28.42377282633889	-11.16674906344178
361 h	-7.14368042867380	30.11855216840471	-13.98425174768991	362 h	-5.94158943898734	31.77566337298172	-11.25945788174735
363 h	-0.56306518644979	31.90540817064116	-16.13871852488397	364 h	-2.31777236751968	33.87887311144614	-13.99076384727631
365 h	-3.88532327303729	32.39127421807807	-16.61665804420769	366 h	2.84976962140003	25.40553892105510	-13.67826701847831
367 h	1.46615155651429	27.41531151674559	-16.00100996107825	368 c	5.56699083131400	27.69430529443050	-15.72467570910764
369 h	-3.93289961973274	24.66411971754158	-12.30792966529819	370 h	-2.63685740169818	22.43850311989393	-10.12894365330014
371 h	-0.69097433008164	23.67173430980921	-12.61436414691627	372 h	8.62250945629585	22.72934651079285	-8.05958555162615
373 h	6.55614185403580	20.31736674748023	-6.95245170395338	374 c	10.43071720388554	19.05759882447839	-7.54696342087725
375 c	-2.58685949203373	18.74352907960922	9.18117174480254	376 h	-4.24253082032923	15.89894856770894	6.73643274891358
377 h	-1.29141115909470	17.08035326798921	5.64319082761711	378 h	-5.43120216092528	46.40157403076491	-12.58508283067544
379 h	-2.21580910145659	47.38118900041341	-13.18076480207662	380 h	-3.86805873115870	48.49156834901311	-10.44641036463370
381 h	-4.90207479685696	38.22717539974620	-15.92989069600365	382 h	-5.98894931529629	36.97736069585297	-13.00034824089084
383 c	-8.96486783891485	38.50429867840445	-15.42514738962907	384 h	-1.84995425340323	44.88481419069325	-16.29376451549665
385 h	-2.66583895511179	41.87122492545297	-17.62451389480200	386 h	0.43106803174943	43.14549798269307	-18.09815482081065
387 h	-8.62972804430775	49.85089217182411	8.11446242805726	388 h	-6.54640051795510	52.24894819294563	7.00943745141216
389 c	-10.41269660534100	53.53517030233025	7.60005818263196	390 c	2.67225643232165	53.84474789397119	-9.09852950185889
391 h	4.24177762666353	56.69528945465407	-6.60524173715126	392 h	1.27657785067297	55.48240797952094	-5.58652230734179
393 h	16.53882378885654	31.67206052080425	-8.01828003192421	394 h	17.54157496288575	32.28920290830764	-11.16728733837210
395 c	14.30917437856956	29.77130747495911	-10.91896793760921	396 h	16.58459229033960	48.75029534947647	1.27068473740889

397 h	17.95840644466239	45.91176821548618	0.01644847953121	398 h	14.83062456001531	46.84008045366988	-0.91664534917414
399 h	11.50595998126052	36.47575421767633	17.70212451903569	400 h	9.37221367108234	38.18924941569087	15.69566577396373
401 h	8.24195251957237	36.79451858077746	18.57438014109533	402 h	-13.35082231127735	26.36898109874454	-3.52240277909205
403 h	-16.45854083911046	27.28901828402988	-4.42848724845930	404 c	-16.21812807370533	25.80835432226637	-0.60290747452683
405 c	6.18612180762971	25.68912002666497	-17.72488131939226	406 h	7.02459406984913	27.67326733870020	-14.20603583088806
407 h	5.66611372062729	29.61299706293527	-16.59325162353463	408 h	10.09568108659767	18.41321757094648	-9.52004347193889
409 h	12.40050793909129	19.79170394587409	-7.44843947916950	410 h	10.32698589085459	17.36421869842272	-6.30038360434453
411 h	-1.65641526251461	17.31403220403712	10.40974261130762	412 h	-1.32394484474581	20.42327890068624	9.12276498903733
413 h	-4.37280484447816	19.32558134913825	10.13142573426941	414 h	-10.32995965066515	38.78775146966379	-13.84572755024103
415 h	-9.23142527365838	40.15198670083142	-16.71360143039885	416 c	-9.59741769127020	36.05810884398235	-16.84637842624982
417 h	-10.07553158512676	54.17680800343297	9.57368513703458	418 h	-12.38762405971508	52.81528114468842	7.49878075873720
419 h	-10.29545278720585	55.22807222771183	6.35406653896262	420 h	1.74952934573827	55.26663335297190	-10.34170733443875
421 h	1.43136710512012	52.14772024023518	-9.07557655109848	422 h	4.48709535774101	53.29157924068971	-10.01064291831400
423 h	15.48236660239762	28.02619727789295	-10.88178458691254	424 h	13.68087937668747	30.06524827977400	-12.90662510089127
425 h	12.58969321577658	29.40819147316414	-9.76510127368144	426 h	-16.65126069796320	23.82743128446637	-1.15959168864293
427 h	-18.00635940838578	26.67621193946135	0.09149168578856	428 h	-14.88242074681645	25.73158287301699	1.02098190136125
429 h	-11.56205366806631	36.09183297235224	-17.59385998083723	430 h	-9.42334260535161	34.37741196522173	-15.59359863332546
431 h	-8.30269866423987	35.76452885387767	-18.47963967388248	432 h	-8.14707749665829	46.58875460487862	18.58206169778615
433 h	-6.21832977904782	48.83226446205330	16.92364251683472	434 h	-4.84995206733353	46.87759528894389	19.33505217857023
435 h	8.08337328809565	26.01084628801530	-18.57200857597362	436 h	4.78065729249988	25.70198929011383	-19.29232854429127
437 h	6.18526979364069	23.75506269584250	-16.89522802758788	438 h	-15.30231880857490	37.25203989411919	10.50729818347892
439 h	-16.13056188727075	41.40794693551602	8.26319308272512	440 c	-16.50714387994599	42.07365418424819	12.30822256349845
441 h	-13.37651086044364	42.70789869052758	9.69705873790586	442 h	-15.34155025221558	42.07307125925556	14.06056115724541
443 h	-17.31333061870735	44.00291660884360	12.08898899351614	444 h	-18.11257054547560	40.74717699602702	12.61820875178239

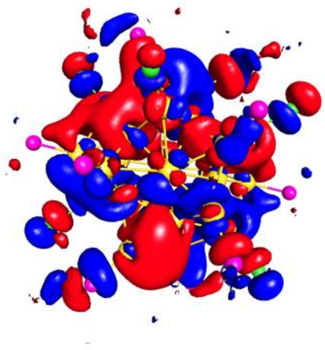

HOMO

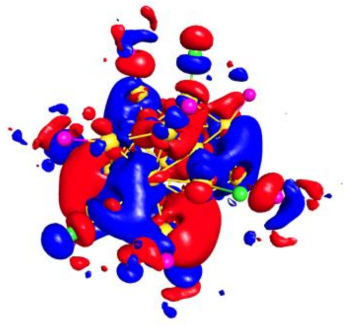

HOMO - 1

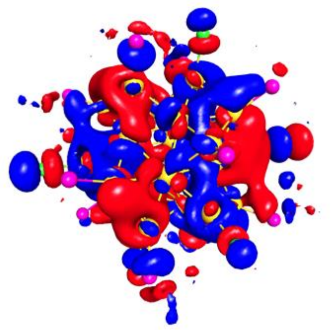

HOMO -2

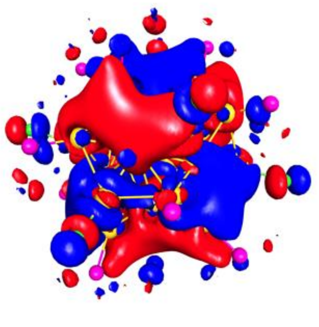

LUMO

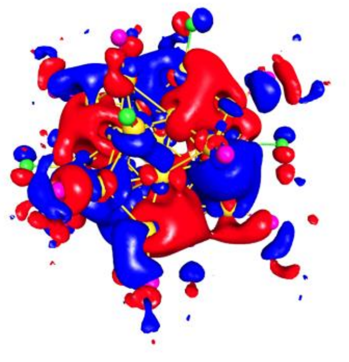

LUMO + 1

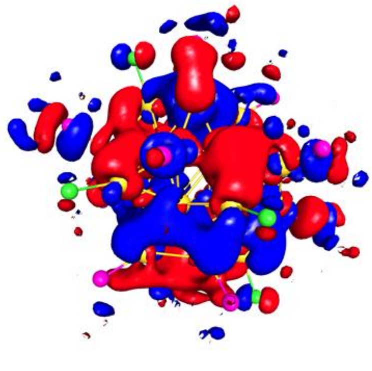

LUMO +2

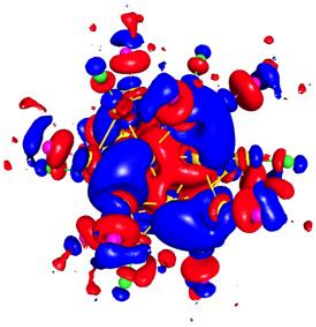

LUMO +3

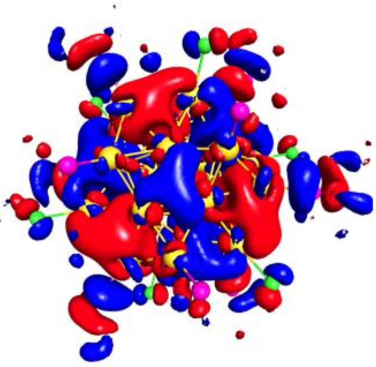

LUMO +4

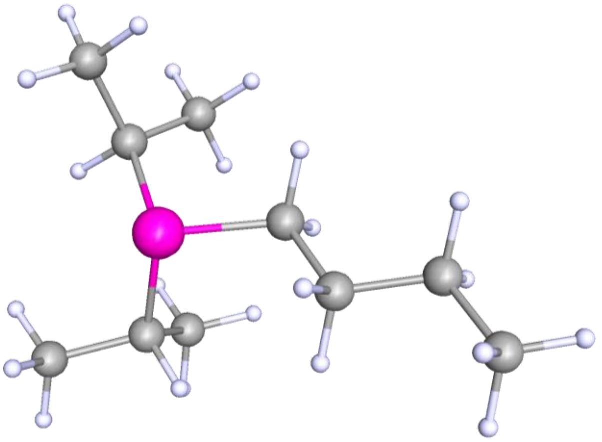

Figure S11: Geometry optimized structure of ${}^{i}Pr_2{}^{n}BuP$

Point group: C_1

Energy: -735.87633553543 H

HOMO-LUMO-Gap: 5.571 eV

1 p	0.11709545658854	0.09893755093767	-0.36154297421865	2 c	-1.59373885260306	1.84357203162367	-3.02554088141821
3 c	-1.33739743580592	1.46647222534433	2.63564235416048	4 c	-1.34113384275355	-3.14335527742134	-0.49591757045569
5 c	-4.23070305022030	1.62544080243955	2.81773080735606	6 h	-0.66415098463353	0.11119183515303	4.10570489949933
7 c	-0.09361899380344	4.03786378282669	3.19892740889408	8 h	-4.81305055724419	2.22501734992729	4.75261740621893
9 h	-5.01243335190364	3.04340310933186	1.47579385020224	10 h	-5.16595391220879	-0.21693001836069	2.43309678713797
11 h	-0.69284474958636	4.76248055108012	5.08342802121485	12 h	2.00327171883752	3.90020737650377	3.18938455740978
13 h	-0.64290906076145	5.49068177020070	1.77673306863508	14 c	0.20570208012788	2.01577746963504	-5.31091044528337
15 h	-1.84444501739771	3.79240397023167	-2.26059163844355	16 c	-4.19777651404623	0.83736994398349	-3.83576036212072
17 h	-4.04979888963780	-1.07636113976710	-4.69377643285238	18 h	-5.04348181392601	2.10086654701301	-5.29528853617998
19 h	-5.55680121305985	0.71812522237679	-2.24272388155593	20 h	2.04599237686377	2.88192504075105	-4.78607615100524
21 h	0.61372248692464	0.10903813163822	-6.10654456590394	22 h	-0.64739422768175	3.16906120836513	-6.85430771468136
23 h	-1.01588549979581	-3.83586215929491	-2.45969289385655	24 h	-3.42906678460234	-3.08564607077327	-0.22689289734091
25 c	-0.13242891734768	-5.00660853176777	1.38235942104148	26 h	1.95909384241360	-5.00087129202240	1.12082568676432
27 h	-0.46443189979385	-4.34968604836388	3.35862884747461	28 c	-1.13344817811793	-7.72486868013517	1.11825166988254
29 h	-0.79136145355114	-8.39523606060934	-0.85129887075026	30 h	-3.22717653042901	-7.72089954563605	1.36683025561873
31 c	0.06268264092922	-9.58240288270495	2.99657182728609	32 h	-0.30733151512621	-9.00475460548516	4.98620619047341
33 h	-0.69950343668362	-11.52605817996574	2.74896107818547	34 h	2.15004635859753	-9.68231244798381	2.74971655229163

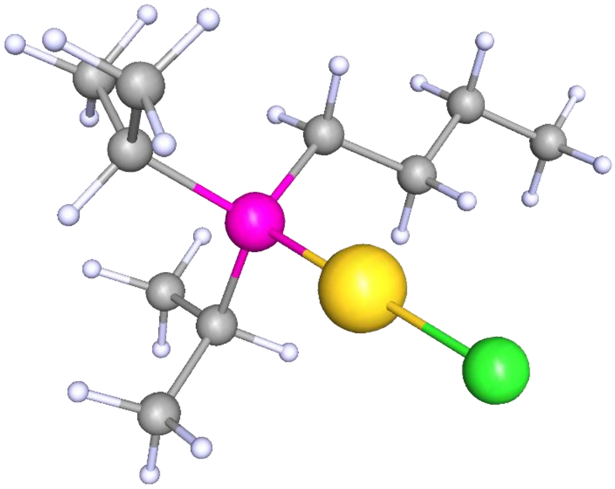

Figure S12: Geometry optimized structure of **1**

Point group: C_1

Energy: -1331.97716043729 H

HOMO-LUMO-Gap: 4.676 eV

1 au	4.36504672798423	-0.05777069010105	-0.24847276666808	2 p	0.03693093003921	-0.01104715359815	-0.29951582367987
3 cl	8.74857843274661	-0.14834827420561	-0.15097830323336	4 c	-1.35635597943654	1.83360038660153	-3.02171036947938
5 c	-1.22868742118166	1.36742820726181	2.73106822925131	6 c	-1.30291429121852	-3.25262350275089	-0.54697970714613
7 c	-4.11777244665515	1.33786556073965	3.04140423157467	8 h	-0.38977540514159	0.06641853102138	4.16096824564868
9 c	-0.11217372568904	4.01479129649957	3.19181217008204	10 h	-4.61879385917849	1.88971808894176	5.00928114991559
11 h	-5.05281973232939	2.71742865378713	1.76155951429052	12 h	-4.95884394272820	-0.55587982729388	2.69611281943271
13 h	-0.63174178646528	4.68405852445019	5.11714114062321	14 h	1.98396765999744	4.00752919369615	3.04805542862451
15 h	-0.85590543040733	5.42626800031184	1.82034625376253	16 c	0.41311364660543	1.73400838045194	-5.33172491847061
17 h	-1.37822141660167	3.81378365276984	-2.30308756577706	18 c	-4.07033363769289	1.06847795158887	-3.74372400762882
19 h	-4.12779560184807	-0.85892205843363	-4.57909831929008	20 h	-4.80728168382352	2.39438135161106	-5.20194007021732
21 h	-5.40161567555404	1.11377100764388	-2.12378573370347	22 h	2.33648439611675	2.43890464167283	-4.87310937082931
23 h	0.61545058383357	-0.22630920415697	-6.06726918162513	24 h	-0.37369468103073	2.91607306948204	-6.88500034329605
25 h	-0.76607180883078	-3.93208817526619	-2.46654406322036	26 h	-3.40357081728940	-3.14908083364587	-0.50421805966556
27 c	-0.31598329643194	-5.10524919353124	1.46588868055992	28 h	1.79046460439098	-5.07303056616779	1.46336540124992
29 h	-0.90828800428950	-4.46340741963483	3.38380272903405	30 c	-1.25222257755626	-7.82712681857165	1.04547187423851
31 h	-0.64218884855401	-8.47477832695816	-0.86508149042273	32 h	-3.36054088709888	-7.84469276446631	1.01727488357473
33 c	-0.28997104871103	-9.68451878667470	3.05305389897609	34 h	-0.92801792181249	-9.13037539386404	4.97999800009214
35 h	-0.99025198445125	-11.63207988731196	2.68836116735908	36 h	1.81173715781854	-9.75767865339673	3.08055111609635

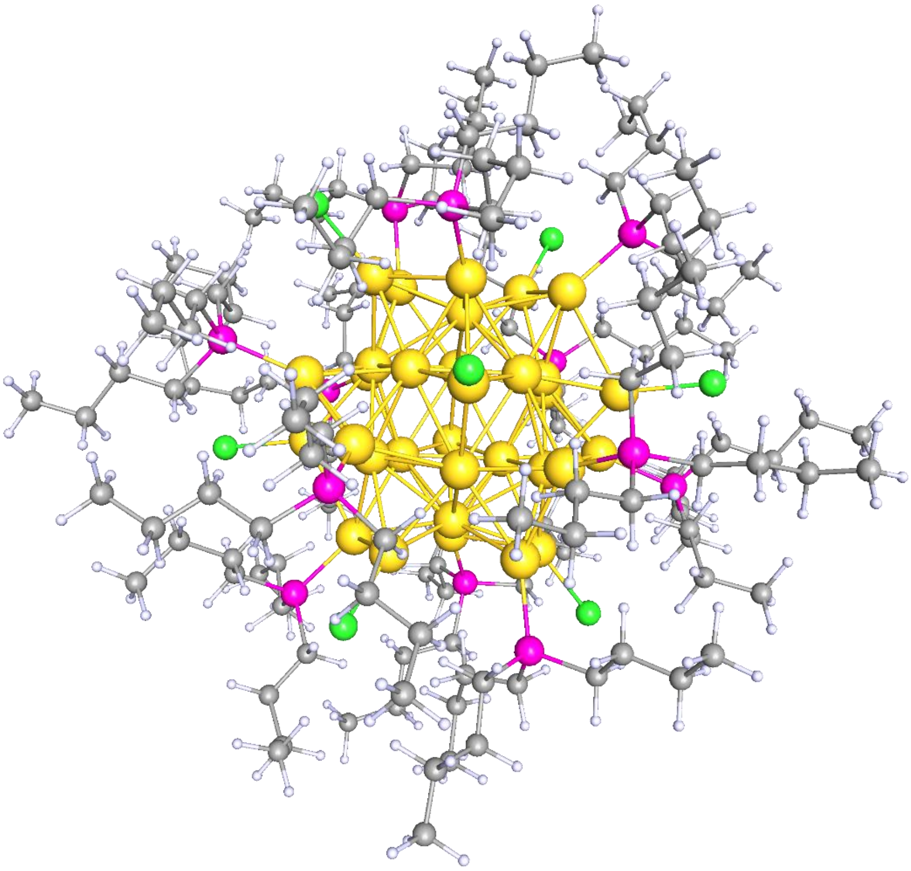

Figure S13: Geometry optimized structure of $Au_{32}(^{n}Bu_3P)_{12}Cl_8$

Point group: C_1

Energy: -17788.91558969258 H

HOMO-LUMO-Gap: 0.488 eV

1 au 24.84359628604379 26.15887918462396 34.37098876549325	2 au 22.90741216584299 27.30086487213529 29.21809111963159
3 au 30.43211561043692 27.33379746065258 34.45174462797550	4 au 26.87814965104154 30.60134050077204 31.67760664384629
5 au 28.69004438665984 22.11744341911229 33.70558821492658	6 au 24.20961365186486 22.10225353555652 30.69615769484829
7 au 28.25191950132082 24.77134225294482 38.46777043494478	8 au 26.39609028194443 30.49667878465694 37.07106788050838
9 au 21.89619390425361 30.58408612619165 33.71503936750112	10 au 19.83153040374952 24.72351841899555 32.61680699058364
11 au 24.02606885574130 20.88430978792290 36.00941108084707	12 au 25.51628046341471 23.70710340191784 25.52392444659026
13 au 27.23680708646400 28.94289769265489 26.28131997165319	14 au 23.32391368699229 32.35899242744523 27.83587583936986
15 au 22.59323794249609 28.14249276283117 23.79285699811080	16 au 20.31602851329224 22.95475409658700 27.11136284555461
17 au 33.02866173852411 23.73568234861131 30.72382519583113	18 au 31.70351485549106 28.93995004322366 29.27335858020576
19 au 33.32464628430920 22.99518038029780 36.22378590758296	20 au 35.62362643120551 28.11177586842968 32.81997913371470
21 au 31.73783069214887 32.34600402410069 33.50030545730390	22 au 29.48207612718574 33.63719902523896 27.75321482268981
23 au 29.06590196124812 20.42969591748129 28.37197903123621	24 au 32.63452591457634 18.71890895186223 32.23089009433284
25 au 26.44370457083098 17.41318286001344 32.25327899452071	26 au 24.19656659193159 18.70292792196424 26.47568585432756
27 cl 27.71897594692039 24.18549443573717 43.02194572948429	28 h 30.66212276984475 18.18037950666137 38.51164628887115
29 p 27.60398711754203 32.51720358238017 40.85442759402095	30 p 17.91453409884424 32.57857287183317 33.72468005550137
31 cl 15.60517695045670 23.93849147314990 34.40972651216255	32 p 21.51889183347637 20.02740441410788 39.59210867633257
33 au 31.06802079174985 24.83793894803787 25.57579177783750	34 au 27.68391622210336 26.27104924338629 21.51558974577372
35 au 29.44611107900882 20.37908892691611 23.01804377180042	36 au 31.88826206031484 30.16938418681492 23.96688789891065
37 cl 21.21432767387487 36.49256953281525 27.35522069658342	38 p 20.61868553753359 30.16849909449699 20.35971968509672
39 p 16.41810718119904 21.13631441748317 26.07491396499517	40 au 33.97772893161076 20.30423341088843 26.27614042708151
41 au 36.04751941494447 26.32898400433491 27.28891716875439	42 p 35.39034555818842 20.99451690917530 39.61598212254933
43 p 39.57658026748975 29.90343372187238 33.70014460374691	44 cl 33.33987517536425 36.46134125771924 34.98951933014622
45 p 29.14599928784045 38.04867112723255 28.39423844662974	46 cl 34.81225324892696 14.71651107182054 33.24724928937770
47 p 26.75497921855875 12.99722839616626 31.64043938817599	48 cl 22.46910041964256 14.58814954203908 25.10384407756767
49 c 31.22718500361474 17.47609417191100 40.41583518346196	50 c 28.38891956974360 35.89192192828344 40.29374871747289
51 c 30.37181926378399 31.13747833858790 42.49106042922037	52 c 25.24508744499771 32.46600136353719 43.45914219746565
53 c 15.74626499135082 31.04616586353036 36.01794367483126	54 c 18.10131704719860 35.97428743241208 34.58895961340652
55 c 16.26856030267309 32.50593932433110 30.61332028039833	56 h 15.86353188771755 28.98513924762376 35.58387079830221
57 c 22.88335836633757 17.70937543353568 41.84781651648906	58 c 20.95816492371438 22.76048344879147 41.69923589646994
59 c 18.36550737818798 18.81898322141603 38.65469739486685	60 cl 28.04102147723050 26.89252439368226 16.94468185921367
61 h 25.47800867807077 32.77894052768756 21.49584270724170	62 p 28.40188757579645 18.28244295170218 19.23064267309219
63 p 34.36705098525212 31.02886624997692 20.35788947887599	64 c 23.04959023559669 31.71610379201433 18.35110203736200

65 c	18.25269332393849	32.56448853782730	21.36819934447016	66 c	18.97787990296106	28.23325033258685	17.92784932721822
67 c	13.76029837091269	23.27816588284947	25.25081421360205	68 c	16.69387603464125	18.88889217950718	23.39386370846117
69 c	15.15260531447918	19.31656450295003	28.78159860819956	70 p	38.06343909634889	18.55604234430562	25.96372262558812
71 cl	40.27297126161003	27.14010470575180	25.47670034765162	72 c	33.01277332086813	19.40746803899631	41.65887688495654
73 c	37.80250313471957	18.65961409816731	38.58935013896277	74 c	37.00742291008228	22.99804190058000	42.00935824128469
75 c	42.20934666632738	27.73222062360920	34.53282822719756	76 c	39.43518005765544	32.26108637992164	36.29494040398811
77 c	40.80966262244625	31.61494502234599	30.91254110350193	78 c	32.17792076376156	39.80350027949840	28.57029761210301
79 c	27.26984965697547	39.54381271401916	25.83810680193858	80 c	27.42729706703821	38.74110025094112	31.38552324056963
81 c	23.72222213882472	11.23481083882175	31.62273052694727	82 c	28.75602450525148	11.53177425537011	34.11602096768284
83 c	28.29161965215469	12.28633483822148	28.55321096596805	84 h	29.45411096826848	17.46386562792626	41.55475837360327
85 c	32.20572041449127	14.74774689091039	40.19279799339620	86 h	29.71079846671766	35.90930903548335	38.65163619819373
87 h	26.61322337210152	36.78765331041902	39.60024860458524	88 c	29.51553881878372	37.39459286397030	42.50472036513987
89 h	30.59557522254964	32.22431663344920	44.28087357698844	90 h	29.80562719835580	29.17513509377381	43.01590701791762
91 c	32.83273066577953	31.10272314015952	40.96290681020970	92 h	24.99756829924559	30.42944101190105	43.93588090782083
93 h	26.15188410679932	33.37543454823369	45.13055613970513	94 c	22.69062236798316	33.70543091177906	42.89295671405397
95 h	16.65635852376132	31.29324974834500	37.89815792725480	96 c	12.96756709929520	31.88671173591075	36.05812584479705
97 h	16.14698220359217	36.74910704698521	34.61818155974154	98 c	19.47343535150083	36.47160462963523	37.09409470728930
99 h	17.79537301068826	32.43607665378934	29.16958717125948	100 h	15.27622816183171	30.64881857350727	30.54520369889821
101 c	14.48113985809044	34.71941386723343	30.02612021409106	102 h	24.65394873789207	18.61346480058255	42.54406388122604
103 h	21.57383141922553	17.58840186054571	43.49496697705160	104 c	23.41886192775653	15.07017428023999	40.78117732226659
105 h	19.79689893547359	22.04671456699119	43.30471122448996	106 h	22.85657340857690	23.26835842224699	42.46678084613621
107 c	19.73295055253927	25.06262137341918	40.43846431743533	108 h	17.61538334845011	20.23049526657483	37.27898076593932
109 h	18.72156827928345	17.06880759868375	37.53996318723463	110 c	16.42935486269166	18.33171691957965	40.75844040779656
111 c	24.89353049862618	33.56498256466637	19.62976172184842	112 c	27.20719027759097	15.03189277692509	19.79769942922758
113 c	25.97131119515857	19.87296190125091	17.28596022661702	114 c	31.04947293051774	17.91792636653735	16.94962944869635
115 c	33.05784164360335	33.44729735312735	18.17345576453814	116 c	34.76867569980478	28.32402001505701	18.17845986689630
117 c	37.58046147279425	32.11018465368466	21.24110553655422	118 h	24.15105934750647	30.08419491855372	17.59371788067927
119 h	22.06910828556264	32.60149135000085	16.71149928050654	120 h	16.82879702967350	31.51004648934551	22.50818555414390
121 h	19.25025992190831	33.80353167095976	22.74529588022157	122 c	16.95081754512813	34.07774647529362	19.25821719298822
123 h	18.46719597556571	29.54452745926803	16.35931107912486	124 h	20.43718613447061	26.92402572534517	17.16784627443597
125 c	16.64628724847004	26.75216846688427	18.79762605942892	126 h	14.11528983147803	23.96163390544551	23.29451659340711
127 h	12.01633861598045	22.09539525710174	25.18148756373640	128 c	13.40663176654730	25.49683961217465	27.09279843758948
129 h	17.47553813672507	20.01556847349470	21.79414485576476	130 h	18.22875560222222	17.55574008138975	23.94733583484372
131 c	14.28834871502486	17.48597263420740	22.56853813413739	132 h	14.68166054039890	20.73739642824487	30.26565910424159
133 h	13.35149930190676	18.42904365511306	28.14575394895185	134 c	16.99034812856748	17.36419781080657	29.88684459046963
135 c	39.97275278793951	20.37860157791950	23.65859442630114	136 c	38.16297800138058	15.21908774505960	24.86695152528929
137 c	39.65825013654793	18.57376712017266	29.08834726690743	138 h	40.04756618970514	22.33948848375518	24.43260166100686
139 h	31.86481632172874	21.01755764715937	42.39163556447973	140 h	34.02446156096529	18.58151456774923	43.31065631092254
141 h	39.14651073790213	19.73758823280961	37.37649576626224	142 h	36.80688178639713	17.35427119338126	37.27090456611430
143 c	39.23658630079230	17.24588113647882	40.68229100699364	144 h	37.55584263758579	21.72199701821149	43.59427814636274
145 h	35.53017253353237	24.29315260315772	42.76106367056237	146 c	39.30552126656710	24.50207533047048	41.08959770247251
147 h	41.91719972338512	27.16252566123990	36.53553818950337	148 h	43.98596912376210	28.86530281491396	34.46856265796379
149 c	42.42291376842869	25.40986099947094	32.79963881674804	150 h	38.73487081259631	31.20739936730842	37.97762403030465
151 h	37.87629660256709	33.57618623933804	35.76882645056790	152 c	41.88393843464398	33.68522342794671	36.93639444089308
153 h	41.18860448485508	30.15794062535750	29.43677552388953	154 h	42.65616831099540	32.45549519702087	31.47711793455053
155 c	39.00077175980619	33.60971705995103	29.83520930303489	156 h	33.21620843422884	38.98440182115544	30.20994695551446
157 h	31.73861605314041	41.80382204271621	29.05563449693960	158 c	33.76596800458023	39.68707355501308	26.14721341199159
159 h	25.49110884687457	38.41636005255414	25.77415573203880	160 h	28.30443472652786	39.13802168527355	24.05071643777508
161 c	26.63214111627894	42.36598879864446	26.04101130093719	162 h	27.86688869641367	37.14237444762243	32.67698855091496
163 h	25.39021835551784	38.56300765016650	30.87687242961044	164 c	28.00617278023791	41.25789875124114	32.71232150692509
165 h	22.64083830420109	11.98478565086982	29.97805356148989	166 h	24.15382968774398	9.21589715337053	31.21191677904769
167 c	22.18856351155556	11.45337575907853	34.07316650793341	168 h	30.49194304329719	12.72572514269566	34.17441218709936
169 h	27.74790288387201	11.86113511914900	35.93481830927266	170 c	29.50436003104932	8.74282700928327	33.84126996206540
171 h	27.72181906219667	13.85370091744692	27.27418343917565	172 h	30.35616395695810	12.51479728867400	28.90640027308123
173 c	27.67063380996752	9.73991933288124	27.30302097650941	174 c	32.67414810902893	13.40439887636040	42.71651615205308
175 h	30.77938414716899	13.65318015559478	39.10166565929144	176 h	33.93932846297248	14.68481505197690	39.00688170584041
177 h	28.20206628621711	37.39338471842107	44.15471456414453	178 h	31.28910086333608	36.46714256762181	43.16032652402341
179 c	30.12986893865768	40.13739494087395	41.76693918552930	180 h	32.60894901189529	29.75590465919642	39.35886825816205
181 h	33.20951999890100	32.97821060359453	40.08176255281016	182 c	35.12420551771134	30.33255514589092	42.57767534710971
183 h	22.97702758025174	35.68469916862642	42.22667012717162	184 h	21.76308843838868	32.68192557773149	41.30500346346559
185 c	20.93656826598534	33.73977154330876	45.20886972641493	186 h	12.13422276596896	31.57640540472657	34.15243175515184
187 h	12.81745846719530	33.95370749571129	36.44879092691301	188 c	11.36060270869462	30.42335379580456	37.99824428618184
189 c	19.61773959190971	39.28528921887850	37.81982022373221	190 h	21.42146747895334	35.68348251578103	36.95698614723317
191 h	18.53001252376977	35.41814652772193	38.65616594566335	192 c	13.02393635930320	34.35106632329087	27.54540830147222
193 h	13.09983136944622	35.01282738062910	31.58748464093932	194 h	15.61904955213974	36.48106582336250	29.87717748708454
195 h	21.64910553046576	14.23591917827621	39.99741310681264	196 h	24.75609544379528	15.23150759058553	39.15975554052886
197 c	24.50217901111712	13.26879022538927	42.78668295003754	198 h	17.92625745299652	24.53747571910684	39.49361482002821
199 h	20.99923883576060	25.74558627475694	38.89936437314581	200 c	19.24920056908581	27.23918513661076	42.30234132114537
201 h	17.16537098137595	16.91036835559251	42.13160529162901	202 h	16.10693832712082	20.10130267895620	41.85439714556468
203 c	13.87917226283731	17.41733697453428	39.70621420744694	204 h	26.64667431720776	33.58178556587315	18.46069051510749
205 c	23.98175116439430	36.30408684984961	19.96768425897562	206 h	25.84537856704344	15.16460318441754	21.40027024241556
207 h	28.85067645765315	13.94922860185868	20.54533359136078	208 c	25.98525996309582	13.64610961067368	17.55973139690492
209 h	25.76090020586610	18.74040381069339	15.52349529240014	210 h	26.77088493619639	21.74938568101379	16.75087629266753
211 c	23.43328806187561	20.23082505378425	18.64204438789818	212 h	31.51482878459107	19.86554311779043	16.30302958385402
213 h	30.30844565757083	16.87656597560498	15.27351746092125	214 c	33.41657869806215	16.61594058942834	18.00032694948764
215 h	31.23218839807226	32.64210905035000	17.49999110345605	216 h	34.33547295920217	33.53498883301749	16.49944078538742
217 c	32.67033478194067	36.09184229391621	19.28987028491280	218 h	35.98113133280065	28.99913991102521	16.59421512119365
219 h	32.84380133841852	27.95864936253692	17.39532166231465	220 c	35.82067282684841	25.90426755735203	19.37426252951179
221 h	38.31336687479515	30.66060720524596	22.58613229613112	222 h	37.30837710134577	33.85823610870231	22.38217844339837
223 c	39.48683664412049	32.55730199640928	19.10139773158816	224 h	15.85440176443202	32.76248477065098	18.03331546496480
225 h	18.39863149868927	34.96848900897692	18.01549783093846	226 c	15.14892525151888	36.15505672972631	20.21965789209981
227 h	15.30216975481636	28.02122143222191	19.80920380969700	228 h	17.24051260869902	25.29557677659218	20.19879609873189
229 c	15.25651493507952	25.49758455612260	16.57324550489219	230 c	10.91404441335928	26.94231387256285	26.69894609465199
231 h	13.47275887678150	24.81065037494726	29.08042138605850	232 h	15.04274544398193	26.81046270259360	26.90231329953121
233 h	12.82072016681957	18.88183785910269	21.99365806061963	234 h	13.48240046129859	16.41509146066444	24.19440213917875
235 c	14.73160850304643	15.63794110455488	20.35896877915021	236 h	17.61242863802477	15.99164662647040	28.41561945320500
237 h	18.73398851983755	18.37072243637474	30.50335325738758	238 c	15.87391346684860	15.93041987543642	32.15521215236351
239 h	38.75862208526582	20.50761447072418	21.94214102198653	240 c	42.63288440991908	19.47345748837611	22.93196999802936
241 h	40.17215281700440	14.71271599351656	24.48735223261513	242 c	36.97538539702174	13.32650230948914	26.71382784000561
243 h	37.16901999266884	15.18484714717508	23.01135030787446	244 h	38.23889059330848	17.70397788867359	30.38127284478776
245 h	39.76693090968995	20.59979363798632	29.65037470842419	246 c	42.22603547168637	17.24112424027178	29.38801595262159

247 h	40.29482558582790	18.63077570737185	41.86337387298769	248 h	37.87380871184813	16.29106474438979	41.97415222445949
249 c	41.12119615482538	15.26275090992028	39.67933950234204	250 h	40.65577290214873	23.23510477199429	40.08385060333418
251 h	38.67190917557097	25.92429028670158	39.67090907415688	252 c	40.70596252680595	25.81450761037132	43.27294555312665
253 c	44.89405093183050	23.91668237082482	33.15460689522314	254 h	42.28099058734126	25.99372530711462	30.78301027231866
255 h	40.76406654057813	24.15527672311379	33.13882173884907	256 h	43.37656986825009	32.30655249843891	37.48851453703271
257 h	42.60480172115052	34.68706467500253	35.22900315256089	258 c	41.56504850811000	35.62467299717783	39.08770362921783
259 h	38.41079164956836	34.98182762501469	31.32124083753412	260 h	37.23793233056367	32.63867308851813	29.21637311985353
261 c	40.13900166550310	35.04881202034970	27.58096783705930	262 h	32.63846421723177	40.38277773168731	24.50825047436021
263 h	34.23753504754472	37.67771826730703	25.71783199204480	264 c	36.20321955335017	41.25819749196187	26.32570301593547
265 h	25.54745263217730	42.71435898210964	27.81047443904579	266 h	28.38888977608001	43.52415898408695	26.17746327648027
267 c	25.04120533753233	43.29104552120927	23.79124854971226	268 h	29.98297333299679	41.20791188789239	33.43029661556837
269 h	27.88526175921896	42.87088570083748	31.36447225866355	270 c	26.20703238297161	41.76755637937939	34.93168304189513
271 h	23.36269678613135	10.86483491647688	35.72108702540852	272 h	21.69380079849379	13.47528265239776	34.40545707750088
273 c	19.77640544512481	9.83455337768118	34.02903026188317	274 h	30.58487419258157	8.48337419166330	32.05282397591345
275 h	27.79754040649596	7.51341993024162	33.69399257886877	276 c	31.15962769417702	7.83726373637616	36.05205231371050
277 h	25.61922994764893	9.69300470505057	26.84238155444589	278 h	28.04078013424104	8.14070244202621	28.62138564545223
279 c	29.17002997293399	9.31024797889074	24.85473098472563	280 h	33.28012638662559	11.41455627158415	42.40673043949172
281 h	30.93259001776999	13.36218575289533	43.89978499585364	282 h	34.17895671746771	14.33230123060271	43.85834045910286
283 h	31.39253611173100	40.11094445139791	40.08141430528082	284 h	28.36098917091145	41.10261390965523	41.15024512690434
285 c	31.36651133563768	41.64773995919361	43.90359475035420	286 h	36.71736371173346	29.87137897822837	41.28239422181668
287 h	34.67317827679329	28.54683330317088	43.60150767393265	288 c	36.01361315613740	32.34211181218286	44.46648182931097
289 h	21.88047732293229	34.79868241746006	46.76836977006764	290 h	20.69363299054741	31.77059430874673	45.91625052719422
291 c	18.33822646411905	34.90367326831232	44.68703384957019	292 h	9.33410766153213	30.87534330501422	37.64606610289057
293 h	11.57727005214502	28.36101038142613	37.62761288877275	294 c	11.98130338053846	30.99810906422704	40.76792976926027
295 h	20.54344245066926	40.35110213442653	36.25647738193607	296 h	20.90544367196712	39.46666549225558	39.47797961669645
297 c	17.07429415369510	40.51116113567890	38.46823544952757	298 h	14.40642153025019	34.01056528530365	25.99357512242614
299 h	11.84981281783532	32.60763856315227	27.68010778282884	300 c	11.33881938035609	36.60523656468009	26.87041277654370
301 h	26.28009118800754	14.09421282533936	43.56089944632488	302 h	23.16275508654705	13.16467194775138	44.41114451997851
303 c	25.02388566034094	10.59886621796952	41.79984699453519	304 h	21.06782385471662	27.76344902356124	43.22828521069895
305 h	18.65811479971195	28.92499883909481	41.18655681066994	306 c	17.24492024624262	26.69684893763636	44.32393192321969
307 h	13.17413874314616	18.84347851375953	38.32605345147028	308 h	14.18244212351558	15.64469852897553	38.60490626712008
309 c	11.88990074838058	16.96055713883338	41.75797527040376	310 c	23.52176285347659	37.75675231426845	17.50360793083357
311 h	25.44441161827665	37.32218685865176	21.08173351201038	312 h	22.25955163177769	36.36363308459415	21.17171412895688
313 h	27.31281711625940	13.53737891513770	15.92424156128925	314 h	24.31195108205102	14.73377167767442	16.88771658240303
315 c	25.11346655926599	10.96555477283473	18.26877414576590	316 h	23.72308449879410	21.55110869684707	20.25802135589193
317 h	22.77100773837116	18.42010960579071	19.49262904129745	318 c	21.36016486375473	21.28293514948517	16.89897946722336
319 h	32.91655081413008	14.72071758631438	18.77593410239839	320 h	34.14678732974411	17.73481904031503	19.63032531991352
321 c	35.49651528277432	16.29282986117940	15.99823694124648	322 h	34.48682796340638	36.81972506212846	20.07289834813053
323 h	31.33913172309541	35.97349390911941	20.91990845562365	324 c	31.66968983845178	37.98018168703521	17.32135759951428
325 h	37.68118529161821	26.26062568053759	20.29479337663081	326 h	34.52376475594580	25.29848203492593	20.92081000559169
327 c	36.07607237768716	23.73596446772304	17.45440178895626	328 h	38.76375637282398	34.00410220921335	17.74791895193212
329 h	39.74057776787470	30.78492613288088	17.99196644587894	330 c	42.08108273292994	33.39894953241291	20.10584434395410
331 h	14.06310276791607	36.88196560301066	18.56753025660999	332 h	13.72169780600288	35.28804528286995	21.50552760937831
333 c	16.44550930726776	38.37625513481451	21.55365011991670	334 h	16.61987614010219	24.31615210618632	15.48584738228597
335 h	14.61517340715941	27.00214846091437	15.24377757643128	336 c	12.99062702849907	23.87712985387249	17.35703169209997
337 h	10.76050476642188	28.39313608805870	28.21758686504534	338 h	9.29608727160999	25.62599453476274	27.00725018754294
339 c	10.63065024456069	28.23117619427454	24.12107880571754	340 h	12.85291887846843	15.03950274839104	19.61790868255527
341 h	15.66757290767591	16.67666501453123	18.77888404739938	342 c	16.27952588252832	13.28555880230332	21.03939152294546
343 h	15.21924500173444	17.32776411137916	33.58785575861669	344 h	17.43508655915979	14.85274903186638	33.06509440143303
345 c	13.72436346030059	14.09838509341549	31.51040733433971	346 h	43.83690166807084	19.30984356727918	24.64882699363537
347 h	42.55662527243576	17.54742885974379	22.07826637690307	348 c	43.91070455881353	21.29674434437390	21.06266755868037
349 h	37.92644425642877	13.45412718538386	28.58432921945393	350 h	34.97433738073877	13.86234915677303	27.08608327181643
351 c	37.10771688469355	10.57945323128225	25.78312235359032	352 c	42.81193200201875	16.61728370218894	32.16443168810967
353 h	43.76423295647599	18.44445645603756	28.60157748536843	354 h	42.26896088899467	15.44840917439960	28.28374437884889
355 h	42.34675774089277	14.65965577885768	41.28288139324387	356 h	42.40783930816474	16.18620557902548	38.28652072160385
357 c	39.90353152719307	12.92266860829668	38.48243609963620	358 h	39.34260633521762	26.99993338619660	44.35491521105520
359 h	41.38338598247378	24.34303585348368	44.62154609524259	360 c	42.94231553687521	27.44608768914019	42.42881647783032
361 h	44.90924307396272	22.34632037174459	31.75156946988053	362 h	46.52444183253102	25.15157053958936	32.64277027464717
363 c	45.31182548060388	22.82947496758046	35.80640444431607	364 h	43.48275918702106	36.26415753123282	39.67954969947322
365 h	40.73938622211082	34.65340647356195	40.76831921923241	366 c	39.95942350498786	37.93735708226786	38.40455779986408
367 h	40.78931250582186	33.65485092510434	26.14403674385316	368 h	38.59260736748580	36.14631368435523	26.66950616757481
369 c	42.30142505642244	36.85673884507175	28.25049304676345	370 h	35.70559536387017	43.24076765384709	26.84021604021330
371 h	37.38073828335317	40.53237683841049	27.91422041418641	372 c	37.75285019366425	41.26414370685092	23.87994932955837
373 h	23.29134599418653	42.12352272207531	23.68350876859060	374 h	26.09516658400555	42.92846703052522	22.00088081412511
375 c	24.34381098564334	46.09504006280201	23.95713598146429	376 h	26.30084572450835	40.14639148717688	36.27455203800947
377 h	24.22502750189676	41.81290201122253	34.21633697258522	378 c	26.79172899125870	44.23011855810598	36.33495090822140
379 h	20.30469607350628	7.82809010226766	33.65773251893290	380 h	18.57651860482908	10.42180105162848	32.40031747970357
381 c	18.23963873098008	9.98424974444725	36.47794883453603	382 h	32.84769372344446	9.09007823399598	36.17829867562899
383 h	30.10340213449503	8.09520947570013	37.85930247174534	384 c	31.99771710170999	5.07872876358839	35.79896521724068
385 h	28.76729479993721	10.89895822685552	23.53413255008749	386 h	31.23318500389829	9.39850324875139	25.26983779274604
387 c	28.54497772603640	6.79358733102222	23.56922665425944	388 h	31.77933874801924	43.62051436345114	43.30716571218053
389 h	30.12435168070505	41.74388413910066	45.60016905827392	390 h	33.18479570286992	40.77401005625705	44.50272211230389
391 h	37.70044991179309	31.68802710639999	45.53954271750036	392 h	36.53953398147203	34.12480482495506	43.47843159104804
393 h	34.53080897562118	32.81683767081851	45.88062454360794	394 h	17.15249675267822	34.93748375782853	46.42219741946357
395 h	18.50631713061897	36.88382179340644	43.99363571395139	396 h	17.28685729644448	33.81173912195175	43.22748457399122
397 h	10.69047402457746	29.96075951293536	42.06252047837407	398 h	13.94132552201521	30.43527672952584	41.27831263525501
399 h	11.78076821501672	33.05207397336876	41.18609179280335	400 h	17.32555656977824	42.51219367660209	39.06088140794670
401 h	15.75451700660327	40.51257181983642	36.83121435642936	402 h	16.12296148475182	39.49608323430790	40.04875625609524
403 h	10.28754498730892	36.27124236206392	25.08042306346342	404 h	9.91951380838858	36.98021234970171	28.37863653832427
405 h	12.47753636335097	38.35623918899891	26.62076262492160	406 h	25.78871131287266	9.35721904645029	43.31325787731995
407 h	23.27014387681908	9.69834184482775	41.06260892909872	408 h	26.42883764240273	10.62162771729435	40.23282712077829
409 h	16.89284918062510	28.38914569166201	45.52222425474240	410 h	15.41143056198280	26.15035043536213	43.44637413274954
411 h	17.82044166569382	25.13809138621628	45.61315328732965	412 h	10.07111165823899	16.30676858901226	40.93189557630874
413 h	12.52435757720023	15.49967851887655	43.13395186967816	414 h	11.49620398632848	18.71927630610826	42.84415974686443
415 h	22.96269351406055	39.74547582640975	17.89786220118996	416 h	25.25587204580045	37.80788061024109	16.31015141472515
417 h	21.99026526186291	36.90779420473274	16.33682473858385	418 h	23.88062356558889	11.08896773054160	19.97163166633239
419 h	26.78525166404885	9.82070716218401	18.84683056590465	420 c	23.70623726596628	9.61897530143096	16.12805844421683
421 h	19.73865244809181	21.87629039946912	18.10390844448321	422 h	22.07934775039716	23.03472938895349	15.97478600678977
423 c	20.39865279584274	19.43377652082315	14.88581344669137	424 h	34.74105365874833	15.13821747203409	14.40468237782206
425 h	35.95994560053902	18.17652894761562	15.17547909318795	426 c	37.91099443790026	15.06536930385441	17.01884416945151
427 h	29.84876652733719	37.25912518533753	16.54247554480919	428 h	33.00526420700516	38.04268803927305	15.69165108686212

429 c	31.28887281904056	40.65671394640441	18.35301664075292	430 h	34.21425041779195	23.45119192214884	16.50874671187869
431 h	36.46546835329502	21.96254078879514	18.52132673576708	432 c	38.14430455656489	24.10358088872007	15.45865258563471
433 h	42.77176621240444	31.95253270835277	21.47191234167016	434 h	41.84884111031221	35.17821299357628	21.21340548939680
435 c	44.04286722251816	33.80196482876371	18.01659687086921	436 h	15.03432587247264	39.82633729208116	22.12458399791549
437 h	17.48008556994696	37.78976191381242	23.28817772309458	438 h	17.83438752754727	39.31382116849266	20.27937347664106
439 h	12.00980713623386	23.07482758529040	15.68055000068189	440 h	13.58155514542241	22.26300597973862	18.57093148407243
441 h	11.57841381848806	24.99990654035880	18.44033388633906	442 h	8.85617007243164	29.35373120810385	24.02130489647798
443 h	12.23271046662523	29.54666372956576	23.75107156773986	444 h	10.57742260606189	26.83960394746672	22.54480935740967
445 h	16.51853015344255	12.03583809756340	19.36495195256493	446 h	18.19699569772903	13.75824977142595	21.76165454142220
447 h	15.31404135906415	12.15928307295206	22.53293147364852	448 h	13.08002417756802	13.05391827366312	33.21861619357491
449 h	12.04332132364600	15.08963614511154	30.72520998477661	450 h	14.34055030535742	12.67265870219155	30.08862301498413
451 h	43.92714661953224	23.22586589217991	21.90675437232663	452 h	42.72639569829912	21.44253352375492	19.32403871233105
453 c	46.59848343179261	20.50089441360862	20.34323624707502	454 h	35.92037110923987	10.36583200455505	24.05292130829485
455 h	39.08093002439083	10.13785508802643	25.18345069089885	456 c	36.27504530245346	8.66990463077276	27.79572640796621
457 h	41.30275203313212	15.34975204255574	32.90546211142640	458 h	42.68653670924390	18.38192831965027	33.30761159766137
459 c	45.40782214240308	15.39753575534374	32.54569297549849	460 h	41.37364708748752	11.56475230700248	37.83732647121440
461 h	38.70741822892719	13.39918037759593	36.82009515300869	462 h	38.68094164538731	11.91762989638529	39.87070184219144
463 h	43.93524135435960	28.29219588699623	44.07647880003879	464 h	42.31687765994378	29.02976754318137	41.19213671194311
465 h	44.35375242011533	26.31900687030553	41.34870488022374	466 h	47.04232729666766	21.63818472524600	35.87625844850786
467 h	43.69061729122375	21.62383495799693	36.40112686067327	468 h	45.53363262498866	24.33966611652670	37.25347515384954
469 h	39.84755464733816	39.27476562980423	40.02309723527490	470 h	37.99220105378357	37.42888204332159	37.86286034567268
471 h	40.80155199308130	38.98180415020720	36.78216478506918	472 h	42.96336151660228	37.90903631429163	26.55401311410165
473 h	43.96982148658581	35.84479555777723	29.03623093993689	474 h	41.69117376460016	38.27712952200520	29.68041939440276
475 h	39.50377294747592	42.40908454919098	24.08234210768306	476 h	36.64855879862464	42.06625404562948	22.27741820185387
477 h	38.33174247855116	39.31622457489144	23.32991118651646	478 h	23.19029068662036	46.69920483138819	22.30711604432070
479 h	26.05958030190565	47.31270454044464	24.01301403987192	480 h	23.22665282093680	46.50110749041503	25.69334328699848
481 h	25.45817673884133	44.54127506074206	37.93063447855059	482 h	26.64889086467979	45.89452813812472	35.05459642609716
483 h	28.73985696355578	44.20874347049820	37.12823117926730	484 h	16.52004790882455	8.77969493434052	36.38032161235254
485 h	19.37279886222004	9.34393105000878	38.13236222834256	486 h	17.60935803381707	11.95418871655970	36.87166359387721
487 h	33.19050263818252	4.48604219456122	37.42505530811944	488 h	30.34664045050990	3.77610253200475	35.71374865538736
489 h	33.12476662713965	4.78354785808225	34.04655851956590	490 h	29.64914779553413	6.53745606972099	21.79830985267014
491 h	28.97719692700067	5.16219737053231	24.82699624710405	492 h	26.50514697944885	6.68245656575068	23.06709509389934
493 h	23.12208789722484	7.68142071234550	16.69598401546985	494 h	24.90397678419508	9.44236595934625	14.40607230709751
495 h	21.96626289538456	10.66918262859278	15.58081326310238	496 h	18.86651751226990	20.29419306154523	13.72955276641290
497 h	19.62147985916567	17.68808303685574	15.77089491337621	498 h	21.91709506212652	18.84650772716193	13.55478837210436
499 h	39.33627767088591	14.79564030436831	15.49795880004644	500 h	37.51679017436047	13.17792687424197	17.86242248418412
501 h	38.80005836257398	16.24993848481294	18.51401040112296	502 h	30.58754231781560	41.96098869552489	16.86168504923037
503 h	33.08751967521635	41.45260379062626	19.10172045919101	504 h	29.88728219104168	40.68138726574607	19.92277347481213
505 h	38.27717826172196	22.43650565745518	14.18226729470972	506 h	40.02838622934876	24.36472246489913	16.36165287856909
507 h	37.78584073151395	25.78165217461530	14.24274969480211	508 h	43.42289650523674	35.28042626259212	16.65279328522331
509 h	45.89429876305712	34.40521088942680	18.80792438501107	510 h	44.36753305568781	32.03374696777272	16.92301074906478
511 h	47.46345344085569	21.85612543214284	18.98934369754122	512 h	46.62438867047163	18.59889567040927	19.44197876251340
513 h	47.84781201241884	20.41219144376341	22.03434504763711	514 h	36.31846788601767	6.70207061614311	27.05762896228509
515 h	34.32156134135870	9.06644182990198	28.46743099050356	516 h	37.52554045784719	8.75819454450311	29.48401816146198
517 h	45.74798470877845	14.94062470099102	34.56980899159586	518 h	46.95920631189659	16.67130910668475	31.91184411264431
519 h	45.57986708351157	13.60544692749720	31.45569900795296	520 h	19.11850895576428	36.89704311853740	32.99191412467516

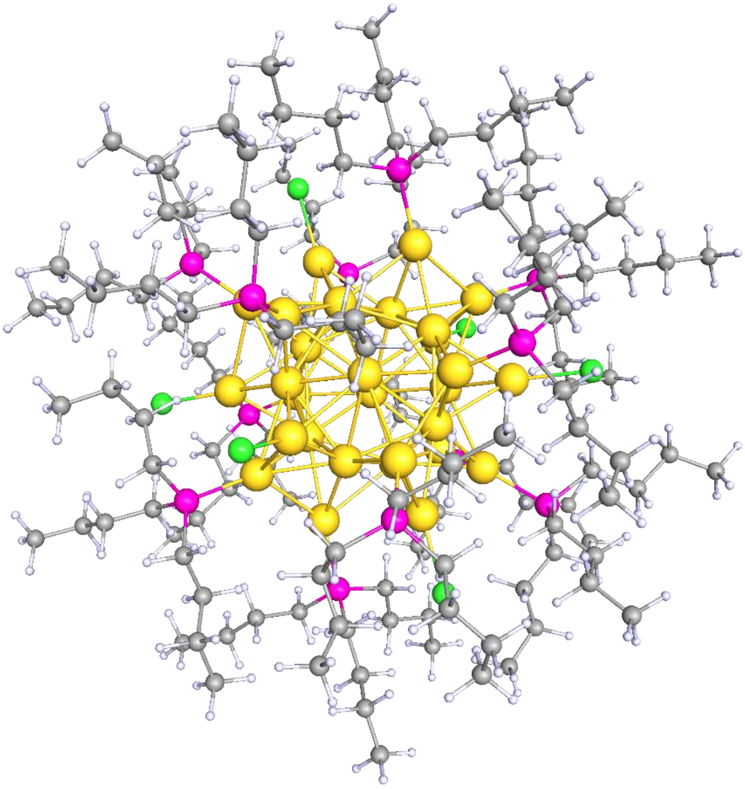

Figure S14: Geometry optimized structure of $Au_{30}(^{n}Bu_3P)_{12}Cl_6$

Point group: C_1

Energy: -16612.75788117461 H

HOMO-LUMO-Gap: 0.855 eV

1 au 25.10204023843793 26.23523233562979 34.24541789912931	2 au 23.02561295218068 27.14070751485702 29.10303838982716
3 au 30.47601903838007 27.41165212283811 34.43536878945745	4 au 27.05245918908325 30.65157472842295 31.51928740159018
5 au 28.67692421713901 21.91510128613939 33.75153900328131	6 au 24.02493532577913 21.97412012986226 30.54347782197746
7 au 27.99683160450979 24.38887140982436 38.37241206863840	8 au 26.65126989279637 30.84843318650414 36.79119440718751
9 au 21.98674610928589 30.51889488043957 33.46650004571170	10 au 20.11251895480966 24.89391607718420 33.06432883190524
11 au 23.89916583167928 20.96226130517021 35.87725948296443	12 au 25.55064736046667 23.76731960330700 25.46189719603053
13 au 27.39582879431847 29.21872372512416 26.24355287157598	14 au 23.24541210334522 32.26558965125505 27.80691049466736
15 au 22.45029999464159 28.01078047710579 23.86717173264374	16 au 20.40965117950301 23.19378389421769 26.36991786933935
17 au 33.01184624508553 23.98539619834166 30.82204197105172	18 au 32.02586129007004 29.10581507631615 29.31757355511655
19 au 33.59105443024276 23.35059497471024 36.13543156885796	20 au 35.62802362063299 28.02658900309748 33.48546457291818
21 au 30.07370216005901 33.77753746502063 27.95097796890287	22 au 28.98579669621929 20.46882319394610 28.54364710172060
23 au 32.89827003302732 18.93260255933919 32.29818673259710	24 au 25.95687070534881 17.32342467479716 32.01284181907708
25 cl 28.01856112582408 24.86727151590218 43.00284610806054	26 h 30.72087371843055 18.31780973135105 38.54462784570852
27 p 27.50259760536987 32.57072532592986 40.86063734311401	28 p 18.05500335446700 32.60150128334133 33.73217563661382
29 cl 15.68559348168943 23.80155339694732 34.19526253495334	30 p 21.44813795416568 19.95681443105544 39.43931069946959
31 au 30.91279605697379 24.78091121501382 25.63211335495917	32 au 27.99594427126458 26.75678200419949 21.57843654780187
33 au 29.26015565943242 20.04228458504218 23.30186808210569	34 au 32.17406268390782 30.12087499291562 24.03413709247572
35 cl 21.47697185253638 36.58971233351953 27.29221501524334	36 p 20.77323243125935 30.23285257123186 20.38419556484671
37 p 16.37278375716428 21.26716790694359 26.05007053216811	38 au 33.92798247110611 20.36148900100668 26.54324397125559
39 au 35.89797984522826 26.09638213738248 26.81670501013107	40 p 35.33702255365586 21.28017702031343 39.66914452481380
41 p 39.65512490406643 30.01191707571822 33.58311022372543	42 p 29.24858902340267 38.17990172928149 28.40344434709390
43 cl 34.93520314911225 14.83980964681814 33.36191755935776	44 p 26.72542982205064 12.90516391003252 31.66441443953273
45 c 31.23155004836028 17.70096417013323 40.49352140898561	46 c 27.85149927315774 36.08164709479185 40.66077879320795
47 c 30.46153159057717 31.43692438625359 42.36251666831310	48 c 25.24141813061406 32.01874525809609 43.50513403075430
49 c 15.82622466252029 30.88791044238398 35.84801718802890	50 c 18.18218544196625 35.91766888636960 34.89832813955836
51 c 16.44442960574269 32.82312230086183 30.60039280060707	52 h 15.97161346455390 28.86382889339008 35.27399531613609
53 c 22.87262668991135 17.63665623329949 41.67226353941544	54 c 20.91219433559221 22.64529984321936 41.62618216548042
55 c 18.26480897519305 18.72011174416194 38.61639466330983	56 cl 27.81542716399957 26.56935612265201 16.91867286496347

57 h	25.63776843650362	32.79376700552851	21.44069222773354	58 p	28.33926398301376	18.09578464278250	19.35179384979936
59 p	34.58617426210768	30.91713133948216	20.38165595914808	60 c	23.23510185971429	31.63506855430339	18.30123103205349
61 c	18.56458894733933	32.78954836814286	21.38616438258054	62 c	18.96465967767167	28.38978253085472	17.98789817682826
63 c	13.62691175282871	23.40566815385727	25.51792046145524	64 c	16.29023974539587	19.05680627844378	23.29575378592021
65 c	15.34507231586985	19.36465267166033	28.80866362359347	66 p	37.93449741466311	18.49189917269015	25.98019665268923
67 cl	40.37817743670909	26.89978358302874	25.62764168679511	68 c	32.94483348221885	19.71239024724394	41.71727149929633
69 c	37.78262880780015	18.92540307688190	38.74003676052483	70 c	36.91812091348847	23.32515710647825	42.06502486852457
71 c	42.37894786665740	27.92578860956698	34.37313889921225	72 c	39.77762754775077	32.52713497947066	36.06013189944733
73 c	40.72485277577356	31.59102663975378	30.64523879153590	74 c	32.10017583834171	40.20322417190073	28.86097622492804
75 c	27.50268886825769	39.56911507626721	25.68949899911994	76 c	27.24009763295469	38.77066712689631	31.24790182457535
77 c	23.85504964238042	10.85794203335666	31.69650382264867	78 c	28.84884361967820	11.64117863989379	34.15746301950792
79 c	28.27614633988563	12.19376044262122	28.56521819880829	80 h	29.42037287570123	17.69448789339851	41.57060309237800
81 c	32.27103101903383	14.98273582056466	40.41986254187539	82 h	29.30587074623433	36.41829736185966	39.17684818504414
83 h	26.04901022100586	36.79920625718599	39.84338258519214	84 c	28.52135033066929	37.51631373532786	43.09673650822227
85 h	30.59775952710849	32.40554761542053	44.22731185844213	86 h	30.12576421642048	29.38912717649911	42.74751208938536
87 c	32.88533864754918	31.79614128560006	40.81318105778675	88 h	25.11482221329634	29.92431620925944	43.68684626964799
89 h	26.18546806192083	32.71430232506940	45.25661411694486	90 c	22.61821126352290	33.22675069309900	43.23814730044224
91 h	16.67350154675671	30.99696563135194	37.77061354135790	92 c	13.03525646811481	31.70659875277211	35.85664025454266
93 h	16.22622110517598	36.68738414320637	34.87507897698338	94 c	19.38983507415139	36.21300500577648	37.52022861952043
95 h	17.99175382502099	32.92121118920662	29.17979025319313	96 h	15.48762401636566	30.96683643730357	30.33025788779922
97 c	14.61995679624914	35.05113914816553	30.18901647993523	98 h	24.68766536933038	18.51147532293687	42.28279899400154
99 h	21.63051732662653	17.57420236674103	43.37386204246622	100 c	23.32282076027049	14.95515107988283	40.66559152512783
101 h	19.90250380584996	21.85942939839199	43.29886047564491	102 h	22.82676815777298	23.24433678807529	42.27648500803139
103 c	19.48334526749472	24.90134207749143	40.49562060933174	104 h	17.45874645887977	20.12135657685831	37.26117374476781
105 h	18.60352867522387	16.97182286745024	37.49368630920267	106 c	16.40569916802448	18.21233226609377	40.79029406400603
107 c	25.14257236273317	33.47297816572170	19.50845716175585	108 c	26.78883966792292	14.95804162164313	19.80382107525416
109 c	26.18652119213738	19.86808265308410	17.22861035900696	110 c	31.05188824932189	17.45917218450835	17.19456441168997
111 c	33.40308556129089	33.49525594343126	18.29476014224386	112 c	34.63777857105465	28.24926784312145	18.10725105620226
113 c	37.93935997281334	31.69599972413021	21.06845604756895	114 h	24.27638298217843	29.94714345692733	17.58350313087770
115 h	22.26656142789550	32.49966071125302	16.64326300497070	116 h	17.11672169868107	31.84829018078766	22.59153870351076
117 h	19.65955165005240	34.01117683553372	22.70456401827161	118 c	17.28638949418301	34.31428019780406	19.26395563857586
119 h	18.51456911356441	29.71256938111663	16.41057155763533	120 h	20.32207657268500	26.98095838578058	17.22090909073077
121 c	16.55163569814764	27.06837137009955	18.90659089496197	122 h	13.77479348752499	24.08696215210316	23.53333432929423
123 h	11.87819160323231	22.23448810681918	25.64098489059713	124 c	13.48049192957803	25.63646303108134	27.38002694190769
125 h	16.78611936846767	20.23012485783931	21.61774702285472	126 h	17.92022544657265	17.75890748581690	23.57792020355303
127 c	13.83159794845304	17.57839916948562	22.82282071265623	128 h	15.15155763470515	20.72112618989591	30.41350444916723
129 h	13.42508969007313	18.61929080442248	28.36832197531379	130 c	17.16923581050690	17.23835497318841	29.56717462322460
131 c	39.75111483402099	20.26986234103445	23.55221969899889	132 c	38.00002399000484	15.14533180457335	24.88975850302633
133 c	39.73952966724141	18.50534629460537	28.99926336023242	134 h	39.98426038683969	22.20619180492894	24.35637550542011
135 h	31.75536525340266	21.32421299947771	42.37649379834831	136 h	33.93636010011269	18.95801024645301	43.41509802637049
137 h	39.14315567489798	19.98532768367864	37.53075609892456	138 h	36.81947277926405	17.58795276254767	37.42932320747585
139 c	39.19137187478690	17.55621194121946	40.88425161412149	140 h	37.41674782675847	22.08766229244827	43.69643653875158
141 h	35.43288956696215	24.65008128100811	42.74740849766231	142 c	39.25720955337358	24.74820063308796	41.16053854356942
143 h	42.15598463301306	27.39613217932943	36.39672558737588	144 h	44.13173462617288	29.08806428225125	34.22604241757421
145 c	42.59921661790590	25.56218074475045	32.69094334665214	146 h	39.41838448539725	31.53528430592295	37.88239799835345
147 h	38.08557309466956	33.72879301812557	35.72614072456388	148 c	42.19442644081676	34.13477325746943	36.23594948548618
149 h	40.87573125202417	30.08738962282194	29.17292887949736	150 h	42.66879875999001	32.30895739414454	31.02122697721331
151 c	38.98060232592731	33.70001587518993	29.67915620698867	152 h	33.11576470559228	39.41052922005282	30.52637125526321
153 h	31.46947307326729	42.13763399861350	29.39617846995608	154 c	33.88016289888974	40.34191405485463	26.57176747149895
155 h	25.78597739552121	38.35937175939986	25.51331607004689	156 h	28.69057668490181	39.19130833339985	23.99253988335838
157 c	26.72237461496233	42.36575574610396	25.80775454320342	158 h	27.68547617305515	37.23005827576234	32.60827674877977
159 h	25.27842525530182	38.38518183028650	30.57776125706500	160 c	27.43647377919081	41.36460392716540	32.54993854544020
161 h	22.69044009944211	11.46303056392005	30.04982226808626	162 h	24.44411191689733	8.87129335607416	31.32916549508533
163 c	22.28072031142211	10.99763595410964	34.13148596362617	164 h	30.54344953632319	12.89765946438103	34.11421768626401
165 h	27.86990075591759	12.04043076974486	35.97901491216582	166 c	29.68441221098655	8.86108204984538	34.02409582726843
167 h	27.57671876482911	13.65949646179265	27.22953780337258	168 h	30.31188231297848	12.63958976928695	28.86963612676760
169 c	27.92236196448872	9.54028814331282	27.42427080154145	170 c	32.66959024365874	13.75837882005463	43.02047065532263
171 h	30.91130345781460	13.81125697084035	39.32270612035440	172 h	34.05145333178455	14.91106899116747	39.30586662438882
173 h	27.05452688583819	37.16344437368062	44.56711738115931	174 h	30.32977128740457	36.78196990044608	43.88787546920656
175 c	28.77258067615663	40.38706587999003	42.68899120049294	176 h	32.76099063199936	30.62297120413023	39.06722974331686
177 h	33.07687558115802	33.79783457640177	40.17424634856093	178 c	35.27378827161530	31.05598604008627	42.30594785815111
179 h	22.80041301661176	35.25894513989048	42.70923194052218	180 h	21.57835618109439	32.30384757122728	41.65957703689261
181 c	21.06145723121884	33.05232526101037	45.69068165309071	182 h	12.27356962064646	31.52959593775206	33.90500073617100
183 h	12.85003952750698	33.73894313586088	36.38868281544398	184 c	11.36345890538398	30.09503681763120	37.62404251998819
185 c	19.49198515519517	38.96666923139153	38.46919761874269	186 h	21.34049105146892	35.42237078212231	37.45282122800572
187 h	18.33978476021666	35.05234777985441	38.93116875754176	188 c	13.22578253078062	34.89626028051645	27.64427903273243
189 h	13.19798395598962	35.16585008028794	31.73693386109975	190 h	15.71786812769137	36.84386149527425	30.23767671352561
191 h	21.51712987246027	14.14046626034726	39.94664938995175	192 h	24.63042007202716	15.03629846530566	39.01571742711313
193 c	24.40460351563316	13.19395944028156	42.71504275094079	194 h	17.67482153537895	24.28530764363798	39.61272324556594
195 h	20.62878037906887	25.71027095924265	38.92581808437657	196 c	18.93717693759332	26.97680561481251	42.46048551439807
197 h	17.20844403409043	16.80645607688701	42.14150982267889	198 h	16.09801396103571	19.98031435462236	41.89193905441717
199 c	13.82846724446468	17.25491321901691	39.83418439120279	200 h	26.92613012881149	33.32666424481902	18.39682423498629
201 c	24.35404850475497	36.27270199822986	19.62128135007073	202 h	25.12163606436719	15.32555321658804	21.03443645939401
203 h	28.11211537665300	13.81849771352504	20.97828470537087	204 c	26.00434122741297	13.49452644346425	17.41949905818579
205 h	26.10262492994566	18.81486469762356	15.40705630929880	206 h	27.11715654445615	21.72455485668316	16.85291869049480
207 c	23.53300754600519	20.33057314772189	18.30712683176501	208 h	31.95319895496416	19.33136726041375	16.86773115635268
209 h	30.26040932062056	16.83910042017743	15.34271570584749	210 c	33.01970986476247	15.54631481194426	18.13691948324221
211 h	31.46703341517524	32.89915273014735	17.72338408298066	212 h	34.58239288396970	33.47003884523937	16.54814108552605
213 c	33.35033270033364	36.16296302490228	19.43130583270275	214 h	35.75388767451560	28.89486087956368	16.44218574409281
215 h	32.64066994874803	28.00571589103772	17.47185347869005	216 c	35.64311599010068	25.73792274114288	19.15461608786226
217 h	38.60766427766337	30.21452307675963	22.41273360192155	218 h	37.89116991613837	33.48354437747275	22.17718250907787
219 c	39.76470412551524	31.92407091219942	18.81949796065620	220 h	16.13369827147953	33.01783360181713	18.07139155300465
221 h	18.74619878277407	35.13849132254385	17.98906654785938	222 c	15.55400709502062	36.46248271373722	20.21020925458340
223 h	15.29544446720552	28.44019674115110	19.89694443231601	224 h	17.06594047440754	25.61084940178630	20.33875737777496
225 c	15.06918872740435	25.85367627201976	16.71466754712097	226 c	10.95700211617536	27.08555154076383	27.24963605830273
227 h	13.75775575758941	24.96013703231812	29.35326727357990	228 h	15.08685309993863	26.94485732774598	27.00063423267493
229 h	12.23052231940694	18.92793607577572	22.61434452021613	230 h	13.38150477443955	16.38634442413720	24.50031731097281
231 c	13.90903569086323	15.87737584125493	20.45400987595073	232 h	17.50810687321437	15.93232163432791	27.94475995612065
233 h	19.03451286845792	18.09090478538414	30.04957984638797	234 c	16.21230357517305	15.69454606884366	31.84377690459343
235 h	38.41793763016444	20.49909511106058	21.93832930598099	236 c	42.30427345587650	19.25022777201178	22.60555631074683
237 h	40.00028702741450	14.66530832402219	24.43722448058943	238 c	36.91012776533557	13.21501603238331	26.76380243508565

239 h	36.94335614138332	15.10191171969057	23.06986570182095	240 h	38.39562282326555	17.68566513099752	30.40098956538379
241 h	39.93722372712871	20.53509726490841	29.51817600608860	242 c	42.29412681271855	17.11492633896228	29.13530369047995
243 h	40.18286725631333	18.97100621746185	42.08765012289498	244 h	37.81662585433602	16.57588798738856	42.14360252971841
245 c	41.15725325122109	15.61426766163589	39.94552585115710	246 h	40.60969329760425	23.47525393659443	40.21313215628138
247 h	38.67331231743994	26.18050771145608	39.69528358355392	248 c	40.63701077646919	26.12619458868341	43.34368268309358
249 c	45.11891603615451	24.13991027104341	33.02902180853819	250 h	42.39959125745335	26.08927281195730	30.66388889824131
251 h	40.98126492245962	24.27780973117764	33.10320552112729	252 h	43.86792597819422	32.87578860592197	36.44319958292777
253 h	42.47527263275508	35.19557748290006	34.43836051702370	254 c	42.19492655465256	36.02858138115502	38.45512594236588
255 h	38.61252412866720	35.12530897353319	31.19093711479289	256 h	37.11361608794975	32.85798766577253	29.18652811675531
257 c	40.07068562606857	35.05613992608424	27.34308343857261	258 h	32.84488906425376	41.12421964749356	24.91266696913816
259 h	34.47990252845079	38.39720805968068	26.02579369415900	260 c	36.22227868363728	41.99279450395531	27.07927667647232
261 h	25.39865031025740	42.64755369854745	27.41844965814492	262 h	28.39300718910998	43.59889275438719	26.17586482759828
263 c	25.40288261210074	43.25152920641255	23.36981472762841	264 h	29.35702870096315	41.60693432097792	33.38128144515034
265 h	27.20915034631150	42.91828451843990	31.14927029836820	266 c	25.45369659543335	41.71241429051098	34.64753057278439
267 h	23.46642815661563	10.45684340575581	35.78587939519762	268 h	21.68953637408239	12.99220309832897	34.46475635481978
269 c	19.94156077679161	9.26898408547357	34.04201892694893	270 h	30.78142176166045	8.54795863417519	32.25508023909144
271 h	28.01636737998554	7.57379917449648	33.93378593493438	272 c	31.35672023947600	8.10971187135876	36.28444454701145
273 h	25.87817632852400	9.19062985779253	27.05987920510018	274 h	28.53081049848468	8.06075565952600	28.79071575464141
275 c	29.38034691615237	9.17611744437262	24.93501380387739	276 h	33.32344419772973	11.76900097954834	42.82788686161425
277 h	30.88582985311254	13.73598654380192	44.13984013048136	278 h	34.11049971014089	14.76858701142886	44.17449371991111
279 h	30.24287719854209	40.74904772966008	41.22275945665591	280 h	26.97051581915899	41.13341631073469	41.89095207012406
281 c	29.43762854948770	41.83721724999352	45.11040010940051	282 h	36.88958345284918	30.97129231620274	40.96072771438553
283 h	35.02907070236157	29.09764827218194	43.03883209185106	284 c	35.96140051081224	32.83836904348885	44.49026151326317
285 h	22.12460334862973	34.00159858275912	47.24482784623236	286 h	20.89302314628944	31.03297373930573	46.26237114082364
287 c	18.42000454290735	34.22545909044915	45.47279293283525	288 h	9.34698455928042	30.52387142489139	37.19032924280202
289 h	11.65041960837577	28.06336021578506	37.14987953443791	290 c	11.82117849356795	30.52343561341153	40.45501134172187
291 h	20.55579181771684	40.13376567957597	37.07546932227122	292 h	20.62969169172027	39.00439155995147	40.24253073311960
293 c	16.90991252391368	40.18457819415589	38.98859281106118	294 h	14.65233472092394	34.79318518168662	26.09949628678729
295 h	12.13054914208790	33.09820738349466	27.55730325798282	296 c	11.44631933424485	37.13856562986374	27.18393496971182
297 h	26.21962418073087	13.99843098724026	43.42095187958207	298 h	23.09862438062404	13.18626270436921	44.37019096340323
299 c	24.83649782007641	10.47047195108758	41.82547655508409	300 h	20.75219829432880	27.57027303438543	43.34856315328650
301 h	18.21054869754245	28.66687562616546	41.43449773855790	302 c	17.03521836003570	26.24223212573332	44.52614972051189
303 h	13.04833474182619	18.66223093628373	38.47573444086818	304 h	14.11708270521333	15.48189828452211	38.73027172095942
305 c	11.91756937689692	16.77404544381325	41.96102938894441	306 c	24.05470141112864	37.55816052383378	17.03672094871710
307 h	25.82118414815493	37.30443665970777	20.71904568739042	308 h	22.58751561613777	36.49120351052025	20.74061848990170
309 h	27.69418742760811	13.11070118671000	16.22106958649556	310 h	24.71288193264663	14.67385391909747	16.24654714373766
311 c	24.68207224206007	10.97222808322713	18.01998299902215	312 h	23.70784311206231	21.41879216013066	20.10315110377276
313 h	22.60123451155946	18.50230104394744	18.79893423486034	314 c	21.81825364310842	21.80665515402261	16.47593375346093
315 h	32.09844517080778	13.68745845549296	18.50797728563435	316 h	33.79959113445111	16.19344744468759	19.98442767338583
317 c	35.17892963866859	15.15673259443392	16.22352239546310	318 h	35.27019903785737	36.69433927728590	20.11962264911918
319 h	32.09568267305099	36.17724065746172	21.12419170551441	320 c	32.45536853107865	38.15257315406014	17.50464341130564
321 h	37.61393086421048	25.95782032300882	19.86471741750158	322 h	34.48483352740951	25.19270078356856	20.82744730871560
323 c	35.53737263928382	23.58718974953308	17.19385851348238	324 h	39.10813733486853	33.40570525610340	17.46976068132906
325 h	39.79387745343021	30.11571308480565	17.74111255934547	326 c	42.48055105657568	32.53825034857538	19.66928124171669
327 h	14.45398356086525	37.18116340592717	18.56273573269108	328 h	14.13140453302882	35.65915614572332	21.54201184353931
329 c	16.93075671372231	38.68118381697013	21.47355897176742	330 h	16.33101259883761	24.51725985981794	15.68443910243971
331 h	14.56768591133375	27.35380346391531	15.32105061509924	332 c	12.65962511778266	24.46937323794285	17.53750856809342
333 h	10.99426803426602	28.58270957109091	28.73072293583856	334 h	9.38679202407357	25.78814322966732	27.79678865815783
335 c	10.35506844258977	28.29837751652763	24.68464294976969	336 h	11.97657761730962	15.09836197887948	20.14641425840093
337 h	14.32478809114831	17.06630506050316	18.76350020190951	338 c	15.79941167665881	13.68086169301241	20.58712964817657
339 h	15.82021581975786	17.01739049253156	33.43255529942905	340 h	17.78126868943515	14.45136460337015	32.49490213617958
341 c	13.86965564107464	14.06294943876645	31.32360530078643	342 h	43.61086354265612	18.94713790513038	24.22558065180729
343 h	42.06102518339068	17.36910703489423	21.68615399740024	344 c	43.56188486427886	21.07805115527909	20.72049354174179
345 h	37.79472241916071	13.45765758645600	28.65490303838892	346 h	34.86664442433843	13.60959416041364	27.07255276290645
347 c	37.28658610627976	10.46431682436843	25.89530379319404	348 c	43.14589389392265	16.63229442661424	31.87539573906799
349 h	43.77896199593349	18.21855399000649	28.13142650418613	350 h	42.17896191554348	15.25914201652866	28.14669205706225
351 h	42.35492441553061	15.05085032470143	41.58533066931165	352 h	42.45682173343383	16.56401314702069	38.58307209576270
353 c	40.03408467551970	13.23415394868645	38.72607787312287	354 h	39.28851473818069	27.40599179118190	44.33355797244214
355 h	41.21415921600488	24.68664873359964	44.77170780078386	356 c	42.96786001691598	27.63364378014634	42.51009242211398
357 h	45.15901017708937	22.55345341421058	31.64557588286903	358 h	46.70781117013852	25.41268689987725	32.47785696683547
359 c	45.60224129483528	23.09514123849982	35.69110394772434	360 h	44.09775721681001	36.92703885143936	38.54489481954526
361 h	41.97272991074670	34.97189340847673	40.26577922181204	362 c	40.17690800426497	38.10609724964220	38.28967296050589
363 h	40.53861074229427	33.60858599701169	25.89057423542023	364 h	38.55175862263373	36.25235330554118	26.50857015550205
365 c	42.39858083577233	36.70976519231454	27.86019402537349	366 h	35.59496385590662	43.88745038835545	27.75940967299764
367 h	37.33007247518512	41.15033244555991	28.66207607034504	368 c	37.93338446121329	42.32317296797059	24.76260487605995
369 h	23.73573492397147	42.01463215445887	23.02013840671410	370 h	26.70099467575431	42.96093004732899	21.73423837973861
371 c	24.56350202303123	46.02514037527149	23.46315207195409	372 h	25.65083348116234	40.15319804437296	36.05105151805030
373 h	23.53339432708758	41.49344679943295	33.81046558141384	374 c	25.64729090831213	44.27229481600942	35.99479444748744
375 h	20.56358436623920	7.28524830596105	33.69120352263312	376 h	18.75343468165577	9.80007717736562	32.38424813185881
377 c	18.33316615363566	9.36391972826996	36.45162298158372	378 h	33.01149419117524	9.40828676788645	36.34959125376516
379 h	30.28663367403971	8.43267162454348	38.07201844861684	380 c	32.26165066685760	5.35757329918631	36.17875707983841
381 h	28.77474629404911	10.65686262371019	23.56398179143699	382 h	31.43019863748483	9.51229502841546	25.27494810120684
383 c	29.00403174209512	6.55080211306214	23.76402895423845	384 h	29.60443951972303	43.90082791405695	44.74698624207316
385 h	27.96682648489157	41.57111201733335	46.59158832702119	386 h	31.26824682008885	41.18442169315654	45.91697109027845
387 h	37.73621571629531	32.22871127751348	45.43944323418220	388 h	36.25673779076370	34.80682585008785	43.80374194169234
389 h	34.46667460805713	32.90041308401253	45.96813964818337	390 h	17.36490823880484	34.08051847958711	47.28522989255752
391 h	18.52453234126017	36.26438522351066	44.96131795914479	392 h	17.27377927505009	33.26249167581198	43.99500635912501
393 h	10.50608956436710	29.36548188166966	41.61675253709564	394 h	13.77348908726001	29.99803945843556	41.03111448378736
395 h	11.52186987626391	32.53996205934602	40.98226846643333	396 h	17.13074915859484	42.12291225729009	39.77283179538039
397 h	15.74774213695280	40.35271453609553	37.24457938496801	398 h	15.80362478947332	39.05971302254427	40.38209884134393
399 h	10.46779500210150	36.97635064692805	25.32994254874410	400 h	9.96437387432800	37.26155347091205	28.67397826778782
401 h	12.49971209494333	38.95954677122214	27.18752212002606	402 h	25.60979110869798	9.27354775724276	43.37081878609150
403 h	23.04397251513765	9.58311884057774	41.17150370771321	404 h	26.20165741499491	10.39317638081855	40.22609208193686
405 h	16.64111687608733	27.86000290075703	45.81081030163939	406 h	15.20113918600581	25.63865434609463	43.68881315338299
407 h	17.73637606226837	24.66281696867134	45.72494100723915	408 h	10.08086269952026	16.08844220482700	41.20157113264751
409 h	12.62268148059425	15.32800715776328	43.31855461565918	410 h	11.53347772795711	18.53008200490427	43.05511748537850
411 h	23.57655282661969	39.59447261383202	17.25375076821776	412 h	25.83258579735250	37.43773259551466	15.91452982868062
413 h	22.53046022676689	36.68853681762906	15.87549599421963	414 h	22.97987463192701	11.35332004584590	19.20316503720559
415 h	25.95578820589257	9.78538247378049	19.20778850298925	416 c	23.90625385856643	9.49159570896054	15.65056825541005
417 h	20.05484276266171	22.30970379910325	17.51089593732286	418 h	22.77540881586896	23.62108269385904	16.00361436785612
419 c	21.11055206666925	20.40252918967651	14.03694966066993	420 h	34.35168172860718	14.61998736760719	14.36000911869144

421 h	36.15695298964553	16.99607057701229	15.90723355809962	422 c	37.10867404812068	13.14917954609266	17.02881650500285
423 h	30.53133927919816	37.63119164702848	16.82125414991431	424 h	33.70643242506839	38.07543425386807	15.80941979819818
425 c	32.41357178642710	40.85828334831176	18.54067748087090	426 h	33.55372373735283	23.41254503656440	16.50618512297536
427 h	35.98325653183657	21.78156794168176	18.18423781635302	428 c	37.33284975013450	23.88457138761915	14.93309823828256
429 h	43.10401200060136	31.06730878764137	21.04079517045513	430 h	42.47765195508877	34.35761593099362	20.73406645267967
431 c	44.36400568886722	32.70373508588637	17.47127050263873	432 h	15.56519820461519	40.17039560291879	22.05663316652639
433 h	17.99738014521777	38.09703127076470	23.18935915189351	434 h	18.30180054438674	39.56955897542466	20.14627138711108
435 h	11.63149662631547	23.67372326307158	15.88530939121312	436 h	13.09540467173560	22.87222704270573	18.83624714784690
437 h	11.33805985967308	25.75905633571139	18.54595334611541	438 h	8.58326058891184	29.42706820836371	24.77272027820657
439 h	11.90019917600279	29.59001283634895	24.07248663086727	440 h	10.09981542698029	26.85890130303923	23.17244645383352
441 h	15.66810896853426	12.46902104059127	18.87452794598515	442 h	17.78771316039386	14.35173305858190	20.72244891547917
443 h	15.42772303161572	12.45653387879305	22.25856450125091	444 h	13.34092409195517	12.93309371354074	33.01641991481826
445 h	12.19159632151704	15.22312517234158	30.81298906169420	446 h	14.21104557588794	12.71181632625571	29.74416257450127
447 h	43.76211210828989	22.96182553407397	21.63885722719026	448 h	42.27003334751318	21.37476446258430	19.08110263219459
449 c	46.14091117472103	20.15479323412709	19.77096368062248	450 h	36.20354664091631	10.12848421138102	24.11681827867821
451 h	39.31335757301545	10.16795783578270	25.39198472310245	452 c	36.51936002650871	8.51807347716936	27.90389447951488
453 h	41.66374906251646	15.50485385096748	32.85760146069253	454 h	43.24639590630152	18.46879923913891	32.90196013962642
455 c	45.70210281458235	15.27650549161926	32.05554246483994	456 h	41.55653835834403	11.91676117251591	38.11798293491623
457 h	38.86221694486540	13.67497171901019	37.03668584714954	458 h	38.81075118836971	12.19668547978944	40.08899962293091
459 h	43.92899399989803	28.53114744501524	44.15038869258331	460 h	42.45007286571509	29.16772319831935	41.16601588413755
461 h	44.37586897652147	26.40129641428283	41.54725090227503	462 h	47.38616832819497	21.98486617984733	35.76174407309733
463 h	44.04000073813205	21.82369236456990	36.30476850288538	464 h	45.76582792134735	24.62697029383997	37.12327202738862
465 h	40.36643058527434	39.46881524469602	39.87905882632167	466 h	38.22664955939182	37.32474017141512	38.36987124417563
467 h	40.35070785185770	39.19628454169459	36.49739048299530	468 h	43.03892890892497	37.68773237078555	26.11224674313276
469 h	44.03004863211615	35.58534142881745	28.56516163948561	470 h	41.98274703774437	38.19302105796088	29.29651604219002
471 h	39.61238904772826	43.50516686790782	25.21222267647522	472 h	36.90588733695074	43.25367362598735	23.17985835122679
473 h	38.63394581387579	40.46967854446827	24.05693771606918	474 h	23.61891179408586	46.60099287133698	21.67546241245155
475 h	26.19939281137258	47.31629380881873	23.76053970423197	476 h	23.20237375489104	46.35842358475321	25.03288683441597
477 h	24.17850643510843	44.46725420870185	37.48627454943845	478 h	25.39583628441466	45.86890222951889	34.64693031355446
479 h	27.52349709450548	44.51574778064946	36.91637956385897	480 h	16.66807724024939	8.08867390946840	36.31209771107984
481 h	19.44454059162364	8.77529873044246	38.13834979368698	482 h	17.61515032937804	11.30793240905232	36.81592913518485
483 h	33.46137393450717	4.88260035092132	37.83847599669168	484 h	30.64278715562861	4.01170076914701	36.16048116571264
485 h	33.40472453793023	4.99471443956032	34.44979537787837	486 h	30.07949963301078	6.34770520592743	21.96917126846662
487 h	29.65611348914608	5.03214629120134	25.06636004495051	488 h	26.97639041171242	6.18435554991283	23.33553199904327
489 h	22.95227161272814	7.68681034861459	16.15174143125236	490 h	25.57663448839286	9.01691095688508	14.46204383453706
491 h	22.57775810841179	10.60136458194628	14.45434149112469	492 h	19.77210504355456	21.53428046739097	12.87434063862978
493 h	20.18191616770876	18.55746330919087	14.45097968727597	494 h	22.78772378108016	20.01282649360107	12.83001813512176
495 h	38.62197654134481	12.92212462005696	15.58768696301715	496 h	36.19720454498349	11.27070808632366	17.29260841374618
497 h	38.03719750604287	13.65711509284137	18.84625789232310	498 h	31.77801301891168	42.22877945630124	17.07885780896426
499 h	34.32149917207656	41.46082605733769	19.19191608367668	500 h	31.10419137014780	41.03034046216443	20.17894760161076
501 h	37.22724605030760	22.21851125228343	13.65313042079537	502 h	39.33210640813350	24.07120117225835	15.56752241126727
503 h	36.87400434302388	25.57837415672299	13.77429250737877	504 h	43.81735506686740	34.20235125705249	16.09756351203496
505 h	46.30256980909965	33.14653461976353	18.15425166547856	506 h	44.46608358685324	30.88753382474151	16.41317020572004
507 h	46.99155306563688	21.51799901394221	18.41502154936811	508 h	45.98392937172851	18.29875793102044	18.78993262654263
509 h	47.49999961818116	19.91308496900294	21.35972653372416	510 h	36.78290934867846	6.55034114270169	27.21090414345072
511 h	34.50334591448535	8.74078694545083	28.46102298663753	512 h	37.66134604667035	8.74760887300495	29.65409193176360
513 h	46.25304505464051	14.94149398691739	34.05656977900532	514 h	47.23697243612433	16.39967095856393	31.15367715824960
515 h	45.64494505325328	13.40475095641977	31.09437271393694	516 h	19.28598348666201	36.97510913200878	33.45034190754661

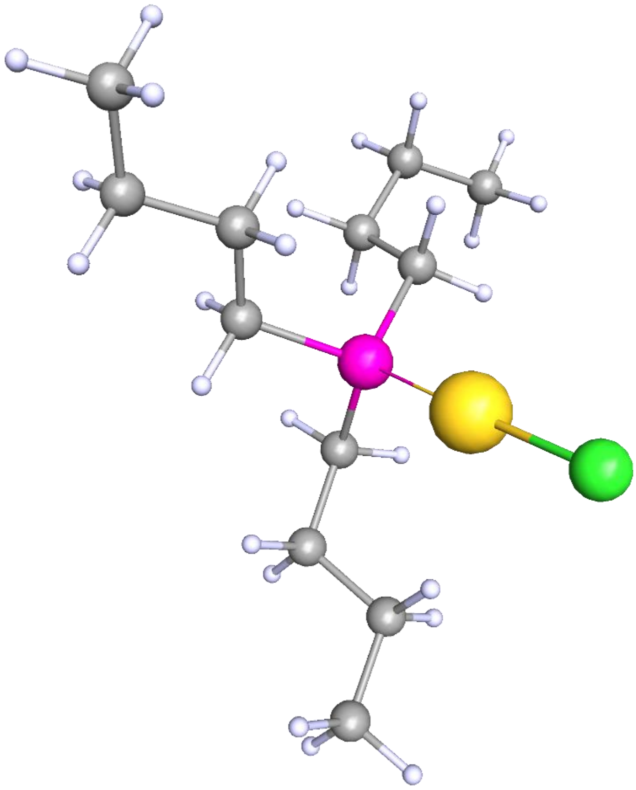

Figure S15: Geometry optimized structure of $^{n}Bu_3PAuCl$

Point group: C_1

Energy: -1410.54298364668 H

HOMO-LUMO-Gap: 4.71 eV

1 au	5.35984158231412	-1.83224507267573	0.82343121059919	2 p	1.10581814270516	-1.12737340454558	0.65959337531162
3 cl	9.66870549872115	-2.60307867963926	1.06588549929142	4 c	-0.04590563174174	1.05776742941543	-1.85269118890492
5 c	-0.08524508189861	0.27622320686930	3.65869372500253	6 c	-0.74781371650270	-4.06600156814004	0.12944064528603
7 c	-2.94358297688180	0.76248326566431	3.88188996925634	8 h	1.01896894830676	2.05144783926536	3.89378061485741
9 c	-3.74782926116221	1.82899557517782	6.47588410044780	10 h	-3.56604351926293	2.09631007513263	2.37465912241984
11 h	-3.99054321415529	-1.03318933577499	3.55084057244724	12 c	0.97670362958723	0.56707725030365	-4.54233161430340
13 h	-2.14670015714342	0.93607142392666	-1.83214935258022	14 c	3.63518576606858	1.62529298024140	-5.04777536369226
15 h	0.94556562158437	-1.49800351289417	-4.96634927854234	16 h	-0.37278446582511	1.43785298093254	-5.90684680660247
17 h	-0.21548197014523	-4.73305423663239	-1.79516391698463	18 h	-2.79373139286210	-3.56987558745871	0.05753935462908
19 c	-0.24283268855072	-6.16076781463981	2.07962611788497	20 h	1.83147782042879	-6.51194308773053	2.19404006075200
21 h	-0.83171194799851	-5.51054612009673	3.99604902180147	22 c	-1.61967341822131	-8.64528957118756	1.45316682145091
23 h	-1.01499693280595	-9.30176626305522	-0.45605748678080	24 h	-3.69107908918385	-8.27861938075030	1.31015612015837
25 c	-1.13351935754004	-10.73899060716615	3.39891991019858	26 h	-1.78325988737946	-10.16975266720187	5.31756779916199
27 h	-2.14546715776392	-12.50774201004599	2.88528162131558	28 h	0.91538262427624	-11.19393591024921	3.53533802814735
29 h	0.57493203748089	-1.00646024718298	5.19161593130263	30 h	-5.85212170475547	1.87424630564404	6.53331183631381
31 h	-3.16008712120570	0.48096561115205	7.98591171345567	32 c	-2.72619789032769	4.47587498360566	7.07990173421691
33 h	-3.28986275718989	5.86777383662108	5.60488534830609	34 h	-0.62866075518592	4.50929749735664	7.21895251781839
35 h	-3.47583452414661	5.16672555891922	8.91746913740205	36 h	3.61671010321805	3.70419547761437	-4.69779634318574
37 c	4.57295430910861	1.09426903905527	-7.73755861261171	38 h	4.99446445478564	0.81250049442407	-3.65584541826366
39 h	3.28469869397423	1.91878848502491	-9.18429767628727	40 h	6.48445066044766	1.90881708566542	-8.04726451697936
41 h	4.70373239764347	-0.97293013039618	-8.10607909089020	42 h	0.45624049567508	3.00564301221397	-1.23021703739084

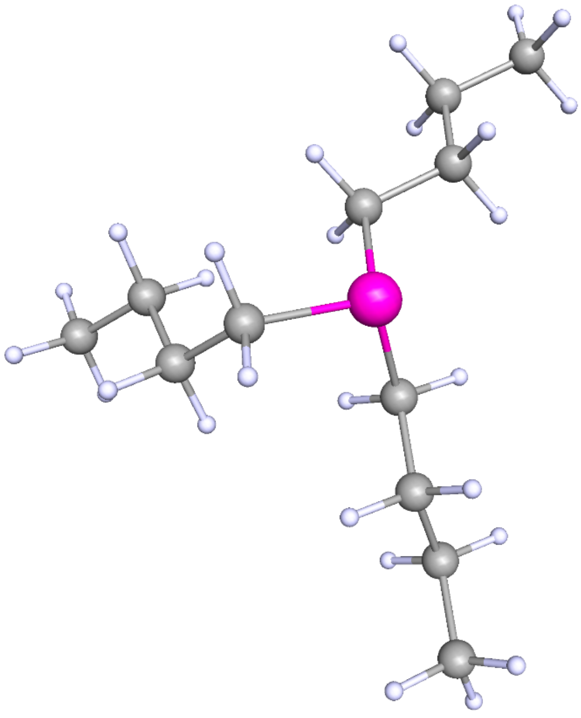

Figure S16: Geometry optimized structure of $^{n}Bu_3P$

Point group: C_1

Energy: -814.44171774973

HOMO-LUMO-Gap: 5.571 eV

1 p	1.08324971646512	-0.71518109412445	0.06702941602759	2 c	-0.55728627093723	1.25887359692706	-2.40885273362709
3 c	0.05280047590567	0.75550067810789	3.15993621326821	4 c	-0.91078335749772	-3.65682810888719	-0.09006861510340
5 c	-2.69908294398483	0.70903184881696	4.11684011582322	6 h	0.76245048327801	2.74041281899702	3.08679044773232
7 h	-2.66324877369267	1.21733047796494	6.16563044226679	8 c	-4.56568846116949	2.49343136482490	2.78370320156902
9 h	-3.45066399592255	-1.25953890348683	4.05477174242161	10 c	0.56436981199425	0.83892779237710	-5.06017184415184
11 h	-2.62999116135323	0.88624112812252	-2.43622892232333	12 h	2.63412273508842	1.23219931629977	-5.00811610612770
13 h	0.38527182958256	-1.19380321317389	-5.59616557807705	14 c	-0.68417031827496	2.46946444404838	-7.11919267992828
15 h	-0.75552358624689	-4.34191796890885	-2.07746643941668	16 h	-2.94665355998844	-3.20457345991993	0.22193097099955
17 c	-0.03880053996891	-5.75177300189502	1.72510868068386	18 h	2.01296103453597	-6.12593986272509	1.41794938758057
19 h	-0.20765031137393	-5.08371366427262	3.71846226477267	20 c	-1.52791365810426	-8.23327408056112	1.43459053418307
21 h	-1.35387086274072	-8.91038548783955	-0.55458086584213	22 h	-3.57966315762766	-7.84969663905572	1.73234251470830
23 c	-0.65879660456568	-10.32127224970545	3.24960398013446	24 h	-0.87529161635240	-9.72961953586964	5.25764608903539
25 h	-1.77235417907994	-12.08483432565737	2.98518415301357	26 h	1.36859839319799	-10.79939884478572	2.95094788875951
27 h	-0.31402361292576	3.27504080599148	-1.84836979278312	28 h	1.27412932489275	-0.19807433872721	4.58673848482138
29 c	-7.17727171325515	2.54600576833424	4.04499312797988	30 h	-3.75859944071823	4.44107232376203	2.75648354018027
31 h	-4.78135252465184	1.93391226739803	0.76858809881370	32 h	-7.05002501193595	3.18669368535858	6.04576404220054
33 h	-8.06229718163879	0.63635957775034	4.05358708539524	34 h	-8.49081840509964	3.84725825834894	3.04437650533863
35 h	-2.75307451161616	2.06528724056312	-7.16452420441622	36 h	-0.50929353801616	4.50227874398275	-6.58702341388002
37 c	0.44269813912442	2.05735025721973	-9.75929369513301	38 h	-0.50173950498114	3.26551421359548	-11.19758629589952
39 h	2.49629712099271	2.51500599328194	-9.79461990823687	40 h	0.23503129436891	0.05607532274901	-10.37648485788198

6. References

[1] Gienger, C., Schynowski, L., Schaefer, J., Schrenk, C., Schnepf, A., *Eur. J. Inorg. Chem.* **2023**, 26, e202200738.

[2] Kenzler, S.; Fetzer, F.; Schrenk, C.; Pollard, N.; Frojd, A.R.; Clayborne, A.Z.; Schnepf. *Angew. Chem. Int. Ed.* **2019**, 58, 5902–5905.

[3] (a) G. M. Sheldrick, *Acta Crystallogr.* **2008**, A64, 112−122. (b) G. M. Sheldrick, *Acta Crystallogr., Sect. C: Struct. Chem.* **2015**, C71, 3−8.

[4] O. V. Dolomanov, L. J. Bourhis, R. J. Gildea, J. A. K. Howard, H. Puschmann, *J. Appl. Crystallogr.* 2009, 42, 339−341.
